# Nanoparticle technology for mRNA: Delivery strategy, clinical application and developmental landscape

**DOI:** 10.7150/thno.84291

**Published:** 2024-01-01

**Authors:** Xiang Li, Jing Qi, Juan Wang, Weiwei Hu, Weichang Zhou, Yi Wang, Tao Li

**Affiliations:** 1 Formulation and Process Development (FPD), WuXi Biologics, 291 Fucheng Road, Hangzhou, 311106, China.; 2 WuXi Biologics, 291 Fucheng Road, Hangzhou, 311106, China.; 3 WuXi Biologics, Waigaoqiao Free Trade Zone, Shanghai, 200131, China.

**Keywords:** mRNA, lipid nanoparticle, lipoplex, vaccine, gene therapy

## Abstract

The mRNA vaccine, a groundbreaking advancement in the field of immunology, has garnered international recognition by being awarded the prestigious Nobel Prize, which has emerged as a promising prophylactic and therapeutic modality for various diseases, especially in cancer, rare disease, and infectious disease such as COVID-19, wherein successful mRNA treatment can be achieved by improving the stability of mRNA and introducing a safe and effective delivery system. Nanotechnology-based delivery systems, such as lipid nanoparticles, lipoplexes, polyplexes, lipid-polymer hybrid nanoparticles and others, have attracted great interest and have been explored for mRNA delivery. Nanoscale platforms can protect mRNA from extracellular degradation while promoting endosome escape after endocytosis, hence improving the efficacy. This review provides an overview of diverse nanoplatforms utilized for mRNA delivery in preclinical and clinical stages, including formulation, preparation process, transfection efficiency, and administration route. Furthermore, the market situation and prospects of mRNA vaccines are discussed here.

## Introduction

Messenger RNA (mRNA) is a naturally occurring molecule that carries the "blueprint" of human cells. It can produce target proteins for therapeutic functions or immunogens that generate immune responses *in vivo* to fight various pathogens. Compared to DNA-based gene therapy, mRNA as a therapeutic modality offers several advantages 1, 2.

Firstly, the transient activity of mRNA allows for time-control, high flexibility, and a wide range of therapeutic effects. Secondly, mRNA can perform vital functions in the cytoplasm without entering the cell nuclei. Thirdly, the likelihood of mRNA integration into the genome is much lower, as antigen translation occurs in the cytoplasm, reducing the risk of insertional mutation and carcinogenesis, and ultimately increasing its safety and clinical applicability. However, mRNA applications have been limited due to perceived instability, poor cell penetration, and insufficient immunostimulation 3-5. Researchers have tried to address these issues by chemically modifying mRNAs, but with little success 6, 7. Therefore, selecting an efficient delivery system to aid in mRNA cell entry and achieve lysosomal escape is of particular importance.

In numerous reports on delivery vectors, viral vectors have been described as an efficient delivery system for mRNA. However, undesired immune responses, toxicity, and vector size are the limiting obstacles to its further development 8. As an important step forward, a variety of non-viral nanovehicles, such as lipid-based nanoparticles, polymeric nanoparticles, lipid-polymer hybrid nanoparticles and others, have attracted considerable attention for mRNA delivery 9-11. Compared with viral vectors, non-viral nanocarriers have several advantages: a) mRNA can be effectively condensed and can avoid being degraded by enzymes 12; b) mRNA can be efficiently targeted and delivered to lymphatic organs such as lymph nodes or antigen-presenting cells, thereby promoting the uptake and presentation of antigens which improves the efficiency of vaccines 13; c) nano-delivery systems induce endosome escape after endocytosis and improve transfection efficiency. Based on these advantages, nano-delivery systems have become a research hotspot for mRNA vaccines and therapeutics. The proton sponge effect is a hypothesis that explains the endosome escape mechanism. After cellular uptake of mRNA-loaded nanoparticles by endocytosis, the pH of the endosomal compartment is acidified from approximately 7.2 to 6.3 in the early endosomes and to around 5.5 in the late endosomes due to the ATP-dependent pump 14, 15. The pKa of nanoparticles is typically designed within the pH change window range of the endosomes, providing them with buffering capacity, which triggers a substantial influx of protons (H^+^) and chloride (Cl^-^) counterions. Subsequently, a large amount of water flows into the endosomes to balance the osmotic pressure, resulting in endosome swelling, rupture, and eventual release of the cargo into the cytosol 16-18. Nanoparticles can collapse during endosome escape or in the cytosol, depending on their material properties, such as pH-sensitive or bioreducible bonds 19, 20, further facilitating mRNA release and transfection.

**Figure 1:**
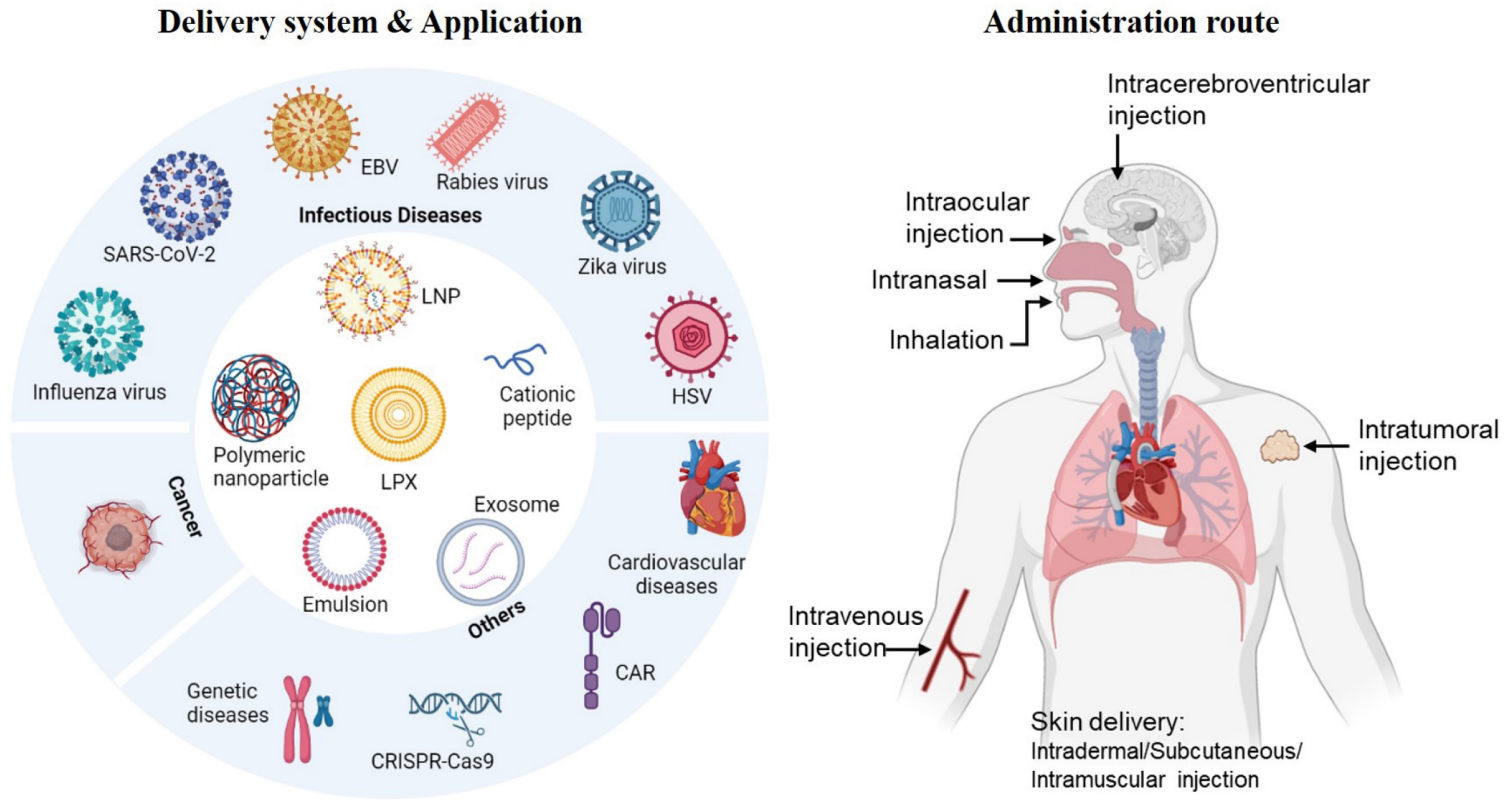
The delivery system, application and administration route of mRNA vaccines. Created by BioRender.com.

In this review, we will summarize recent representative non-viral nanoplatforms used for mRNA delivery. These include lipid-based vehicles such as lipid nanoparticles (LNPs) and lipoplexes (LPX), polymer-based polyplexes, lipid shell coated lipopolyplexes (LPPs), cationic lipid-assisted nanoparticles (CLANs), inorganic nanoparticles, cationic nanoemulsion, *etc.* (**Figure 1**). The formulation, preparation process and application potential of these vectors will be emphasized, as well as an overview of the route of administration. Towards the end, we will provide an analysis of clinical applications and market situation of mRNA vaccines.

## Diverse nanoplatforms for mRNA delivery

### Lipid based nanoparticles

#### Cationic liposomes

**Figure 2:**
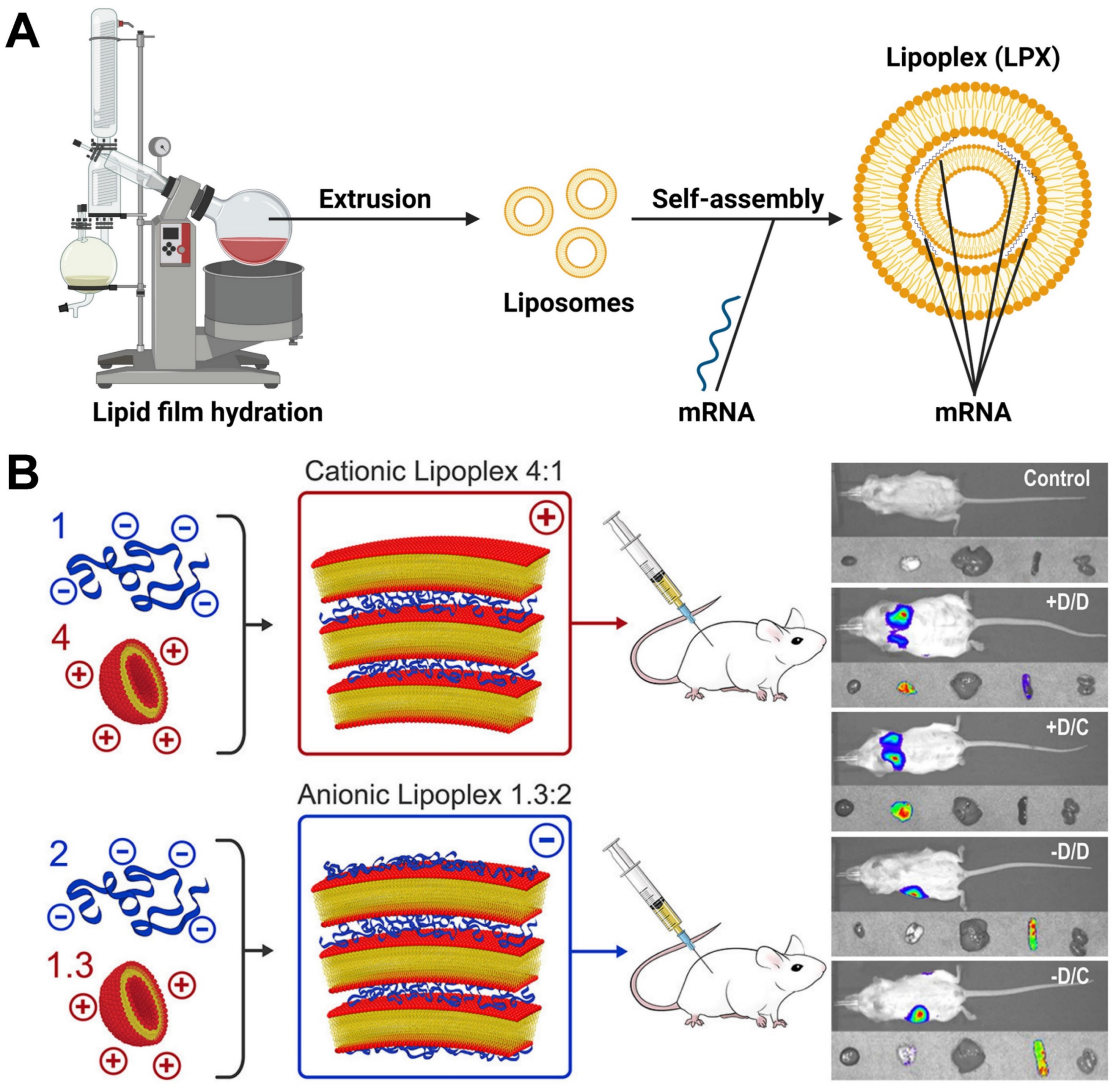
(A) Representative process flow of LPX. Created by BioRender.com. (B) The relationship between charge ratio of LPX and organ targeting. When LPX is positively charged, it is targeted to the mouse lung; when LPX is negatively charged, it is targeted to the mouse spleen. Adapted with permission from 26, copyright 2018 American Chemical Society.

Cationic liposomes are composed of cationic lipids with a positively charged head groups (such as DOTMA or DOTAP) as well as stabilizers such as cholesterol, which spontaneously assemble into vesicles due to their amphiphilic nature 21. In general, the preparation methods of liposomes include thin film dispersion, solvent injection, reverse evaporation and so on 22, 23. When cationic liposomes bind to mRNA, mRNA would be embedded between the lipid bilayer, self-assembling into lipoplex (LPX), which could protect mRNA from nuclease hydrolysis and be taken up by cells easily **(Figure 2A)**. Krajewski *et al.* used 3β-[N-(N',N'-dimethylaminoethane) carbamoyl] (DC-Cholesterol) and DOPE with a ratio of 1:2 to generate cationic liposomes for loading mRNA with dry-film method, which caused high mRNA encapsulation efficiency and successful cell transfection 24. Zhang and colleagues prepared cationic liposomes composed of DOTAP, cholesterol and cholesterol-modified cation peptide DP7 (DP7-C) via thin film dispersion method. The cationic liposomes were found to enhance mRNA incorporation into dendritic cells (DCs) and serve as an immune adjuvant, boosting the immune response. The results confirmed that DOTAP/DP7-C/mRNA liposomes could stimulate DCs maturation and cytokine secretion *in vitro*, demonstrating excellent anti-tumor effect* in vivo* 25. In addition, most of the commercial agents used to deliver mRNA are also cationic liposomes, such as Lipofectin™ (DOTMA:DOPE=1:1(w/w)), MegaFectin™ (DOTMA together with DOPE or cholesterol), TransfectAce™ (dodecyl-trimethylammonium bromide (DDTAB) and DOPE), and Lipofectamine™ (2,3-dioleyloxy-N-[2(sperminecarboxamido)ethyl]-N,Ndimethyl-1-propanaminium trifluoroacetate (DOSPA):DOPE=3:1 (w/w)). Furthermore, Rosigkeit *et al.* explored translation activity of mRNA-LPX *in vivo*. They prepared negatively and positively charged mRNA-LPX by changing the charge ratio between cationic lipid and mRNA. Both of them successfully expressed luciferase *in vivo*. Interestingly, the cationic LPX preferentially targeted the lung, while the anionic LPX was enriched in the spleen **(Figure 2B)** 26. Therefore, the ratio between cationic lipid and mRNA plays a crucial role in determining the organ targeting selectivity of LPX. Researchers further found that the DOTAP/cholesterol (1:1 molar ratio) mRNA-LPX was superior to the DOTAP/DOPE (2:3 molar ratio) mRNA-LPX in transfecting murine bone marrow-derived dendritic cells (BMDCs) 27.

Recently, BioNTech has developed an mRNA-LPX formulation with a charge ratio of 1.3:2 consisting of DOTMA and DOPE, which effectively delivered mRNA to the spleen. It was found that intravenous injection of mRNA-LPX encoding influenza virus hemagglutinin (HA) could induce the maturation of splenic pDC, CD8^+^ and CD8^-^ cDCs and upregulate the activation markers CD40 and CD86 28. Besides, antigen-specific CD8^+^ and CD4^+^ T cells were highly proliferative. The preventive efficacy of the mRNA-LPX vaccine was also assessed in B16-OVA and CT26 models, immunized with OVA-LPX or gp70-LPX, respectively, and the data indicated that mRNA-LPX led to complete and long-lasting protection upon tumor challenge 29.

#### Lipid Nanoparticles (LNPs)

LNPs are the most widely studied non-viral vectors for mRNA delivery due to the commercialization of Onpattro^®^ (patisiran), an siRNA-LNP for the treatment of the polyneuropathy 30, 31. LNPs typically consist of four components, ionizable lipid, phospholipid, cholesterol and PEG-lipid 32. The ionizable lipid component is particularly important in determining the mRNA delivery and transfection efficiency. It is an amphiphilic molecule composed of an ionizable head group and several hydrophobic tails. Ionizable head groups, such as tertiary amines, are uncharged at neutral pH but protonated at lower pH, thereby binding to negatively charged mRNA 33. Once LNPs bind to the cell surface and internalize through hydrophobic interactions or receptor-mediated endocytosis, acidic conditions in the endosome induce cationization of ionizable lipids, transforming to a hexagonal H_II_ structure that break down the endosome membrane and release mRNA into the cytoplasm. At the same time, phospholipids can be degraded *in vivo* due to the presence of ester or amide groups, avoiding the toxic side effects caused by phospholipid accumulation 34.

Phospholipids (DOPE or DSPC) can improve the permeability of cell membrane, facilitating cell uptake and endosome escape 35. Cholesterol plays a crucial role in enhancing particle stability by modulating membrane integrity and rigidity 36. Besides, its derivatives can further affect delivery efficiency and biodistribution of LNPs 37. A study by Sahay *et al.* found that replacing cholesterol with β-sitosterol can facilitate both endosome escape and improve transfection efficiency, as well as increase the stability of LNPs after nebulization 38. PEG-lipids have a significant impact on adjusting particle size and zeta potential, contributing to particle stabilization by reducing particle aggregation, prolonging blood circulation time of nanoparticles by reducing renal and monocyte phagocyte system (MPS)-mediated clearance, and enabling the coupling of specific ligands to particles for targeted delivery 39. Taken together, compared to cationic liposomes, LNPs behave electrically neutral at physiological pH, greatly reducing toxicity and improving safety. They also have a micellar structure within the particle core, providing better kinetic stability and a more rigid morphology than liposomes, placing LNPs as the leading non-viral vector for mRNA delivery.

As microfluidic devices show many advantages, they are preferably chosen to prepare mRNA-loaded LNPs 40, 41. In 2020, there are two LNPs-based vaccines against the SARS-CoV-2 spike protein (mRNA-1273 and BNT162b2) that have been approved for emergency use in the United States and other countries (**Table 1**). Currently, there are the other two mRNA-LNP vaccines, AWcorna and SYS6006, have respectively received Emergency Use Authorization (EUA) in Indonesia and China.

#### Selective organ targeting lipid nanoparticles (SORT-LNPs)

When delivered into the blood, LNPs are primarily taken up by the liver, which limits their utility for broad therapeutic applications. Therefore, achieving extrahepatic targeting of LNPs has become a key area of research. In this regard, Daniel Siegwart's team has proposed SORT-LNPs, which enable achieve selective organ targeting by introducing a fifth component: SORT lipids 42. The study revealed that when using the cationic lipid DOTAP as the SORT molecule, the SORT-LNPs can effectively target the lungs. On the other hand, by utilizing the negatively charged 1,2-dioleoyl-sn-glycero-3-phosphate (18PA) as the SORT molecule, the SORT-LNPs can achieve targeting to the spleen 42. These SORT lipids allow different serum proteins to recognize and bind to them. The adsorbed proteins on the LNP surface then interact with homologous receptors expressed by cells in the target organs, promoting mRNA delivery 43. Recode Therapeutics, utilizing the SORT-LNPs system, has been established and boasts an extensive R&D pipeline. One notable application is the development of an inhaled mRNA vaccine for the treatment of primary ciliary dyskinesia, which is currently undergoing phase I clinical trials (NCT05737485).

#### Lipid nano-crystal (LNC)

**Figure 3:**
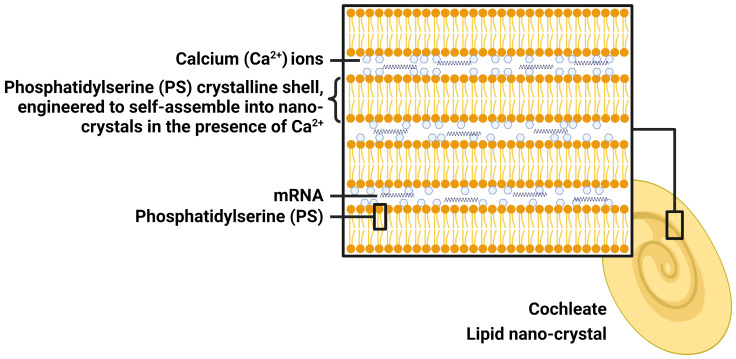
The structure of lipid nano-crystal (LNC). Created by BioRender.com

LNC is prepared using calcium (Ca^2+^) ions and naturally occurring phospholipids (phosphatidylserine, PS), which is a component of the cell membrane. When Ca^2+^ interacts with PS, they self-assemble spontaneously into stable crystalline spiral structures with multiple layers, lacking of internal aqueous space (**Figure 3**). For this reason, LNC will be a promising next generation of mRNA delivery system. mRNA can be trapped within the layers, where it is protected from water and harmful external elements, making LNC particularly suited for oral administration and providing mRNA stability for an extended period of time at room temperature. LNC achieves effective intracellular delivery *via* both phagocytosis and fusion. Endogenous PS, typically confined to the inner layer of the membrane facing the cytosol, moves towards the outer layer (cell membrane) upon cellular infection or inflammation 44. This process acts as a precursor for cell and LNC fusion, thereby facilitating phagocytosis of LNC by cells. Once inside the low-calcium cytoplasm, LNC collapses gradually to release mRNA. The LNC platform has been utilized by Matinas Biopharma to develop two promising drug candidates, and the same LNC technology will also be used to develop oral mRNA vaccines in collaboration with BioNTech. Although early *in vitro* studies showed efficacy, recent* in vivo* studies in mice showed no activity of LNC-based oral mRNA vaccines. Therefore, the cooperation between the two sides ended.

### Polymeric nanoparticles

Cationic polymers have been widely studied for mRNA delivery based on the diversified structure and facile modification. Cationic polymers can bind to the negatively charged mRNA* via* electrostatic interaction, protecting mRNA from RNase, enhancing cell-specific uptake, and promoting lysosomal escape 45. Moreover, mRNA loaded onto polymer can be more effectively transported to the lymph nodes, representing high adjuvanticity and triggering stronger humoral and cellular immune responses. With continuous exploration, various cationic polymers have therefore been investigated for mRNA delivery, including synthetic polymers such as polyethyleneimine (PEI), polyester, dendrimers, and others 46-48, as well as natural polymers such as chitosan 49, 50 (**Table 2**).

#### Polyethyleneimine (PEI)-based nanoparticles

PEI is a classic polymer for mRNA delivery, which can provide high density positive charges for mRNA complexation and endosomal escape owing to the numerous amino groups. However, the strong affinity between mRNA and PEI can impair the transfection process of mRNA 51, 52. Moreover, the inherent disadvantages of PEI, including poor biodegradability and high toxicity, limit its widespread therapeutic applications 53. Reportedly, PEIs with high molecular weight exhibit excellent nucleic acid delivery efficiency but cause serious cytotoxicity, while those with low-molar-mass show minimal toxicity, they are accompanied by a reduced efficiency 54, 55.

To address the toxicity concerns associated with PEI, researchers have undertaken chemical modifications to enhance transfection efficiency while decreasing toxicity. For instance, PSA/mRNA nanoparticles were developed by adding mRNA to PEI_2k_-stearic acid (PSA) with the indicated weight ratio. Subcutaneous administration of the nanoparticles demonstrated efficient mRNA delivery to dendritic cells, resulting in antigen-specific immune responses 56. Additionally, cyclodextrin (CD) and polyethylene glycol (PEG) modification can reduce the charge density of PEI but retain its ability to condense nucleic acids. Li *et al.* achieved successful mRNA delivery using CD-PEI conjugates, in which pre-diluted polymer and mRNA were mixed and vortexed briefly, before incubating at room temperature to obtain CD-PEI/mRNA complexes. The modified PEI could help mRNA to migrate to the lymph nodes and elicit immune responses, while retaining the potent mucosal adjuvant activity 57, 58. Lastly, PEI-*g*-PEG/mRNA nanoparticles (PEG grafting ratio was 0.5%) bearing amino or amino acid terminal groups displayed the highest gene transfection efficiency in the lungs following systemic administration, when the aforementioned mixed preparation method was applied 59. Above all, these PEI-based polymeric nanocarriers are provided with improved transfection efficiency and reduced toxicity.

#### Polyester-based nanoparticles

**Figure 4:**
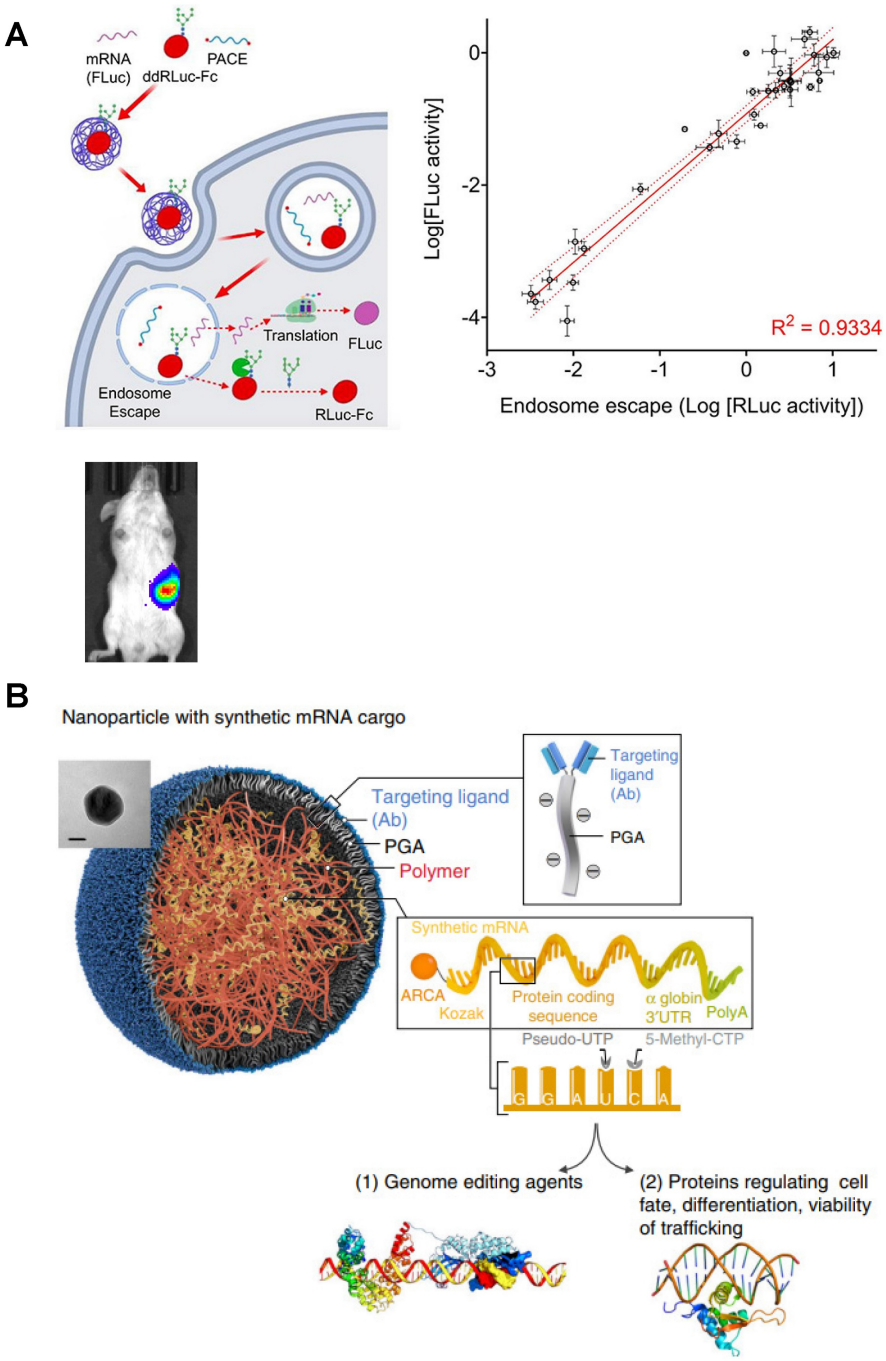
Typical polyesters as mRNA delivery vectors. (A) Schematic representation of mRNA delivery to spleen with PACE. Adapted with permission from 62, copyright 2020 American Chemical Society. (B) Schematic illustration of specific cell types targeted PBAE based nanoparticle coated with ligands through a polyglutamic acid (PGA) linker for mRNA delivery. Adapted with permission from 64, copyright 2017 Nature Portfolio.

Polyesters, including polyhydroxyalkanoate (PHA), poly(amine-co-ester) (PACE), poly (b-amino esters) (PBAE) and others 60, 61, have favorable biodegradability due to the hydrolysis of ester bonds, making them valuable for clinical translation. PACE polymer library with different end groups was synthetized by Jiang *et al.*, and then PACE/mRNA polyplexes were prepared in 25 mM sodium acetate buffer (pH 5.8) by a brief vortex. Both *in vitro* and *in vivo* results indicated that PACE could serve as a platform for spleen-specific mRNA delivery 62 (**Figure 4A**). Apart from PACE, several studies found that chemically modified PBAE can be more effective to promote endosomal escape and enhance transfection efficiency 63. When polycaprolactone (PCL)-based PBAE terpolymer was synthetized to complex mRNA *via* a brief mixing protocol, the transfection efficacy of the prepared polyplexes was several times higher than PEI-based polyplexes, accumulating in the spleen after intravenous administration, and benefitting tumor immunotherapy 64. Furthermore, by using similar mixed preparation protocol, PBAE-mRNA polyplex can be developed into a targeted-like mRNA nanocarrier coated with ligands through a polyglutamic acid (PGA) linker to improve cell targeting and transfection efficiency 65 (**Figure 4B**).

#### 2.2.3 Dendrimers-based nanoparticles

**Figure 5:**
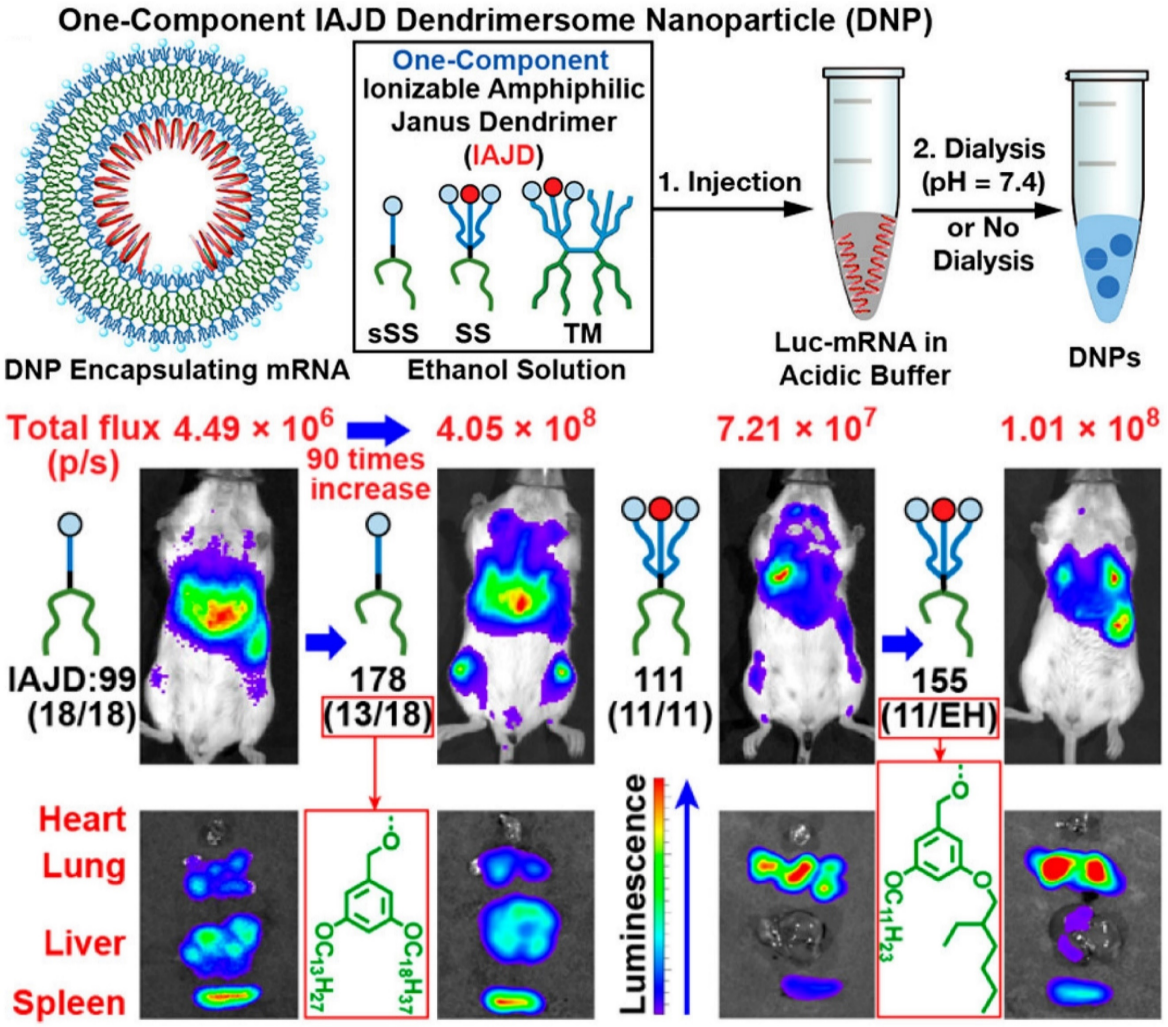
Schematic representation of mRNA targeted delivery to spleen, liver, and lung with designed ionizable amphiphilic Janus dendrimer (IAJD). Adapted with permission from 67, copyright 2022 American Chemical Society.

Polyamide amines (PAMAMs) and branched PEI are widely studied dendrimers for nucleic acid delivery, containing numerous amino groups along their polymer chains, and presenting a “tree-like” architecture. A nano vaccine platform based on alkyl-chain-modified PAMAM dendrimers was constructed by Chahal *et al.* with a microfluidic mixing device, encapsulating antigen-expressing replicon mRNAs to fight against various diseases, such as lethal Ebola, H1N1 influenza, Zika and* Toxoplasma gondii* 66. Moreover, a one-component multifunctional sequence-defined ionizable amphiphilic Janus dendrimer (IAJD) delivery system for mRNA was developed by injecting the ethanol solution of IAJD into the acetate buffer of Luc-mRNA (**Figure 5**). The results confirmed that the targeted delivery of mRNA to spleen, liver, and lung could be attainable by designing the hydrophobic region of IAJDs through alternating dissimilar alkyl lengths 67.

#### 2.2.4 Other polymers-based nanoparticles

Apart from these above-mentioned polymers, other polymer-based vectors have also been explored for mRNA delivery. For example, chitosan-coated PLGA nanoparticles were prepared by emulsion-diffusion-evaporation, which were used to deliver nuclease-encoding mRNA to correct the surfactant protein B (SP-B) deficiency in mice 68. An 8000Da polyacrylic acid (PAA_8k_) grafted with oligoalkylamine has been developed as an mRNA delivery vehicle, with high mRNA transfection efficiency attributed to high buffering capacity in the pH range of 6.2 to 6.5 69. Another intelligent mRNA delivery system based on block copolymer poly(aspartic acid-(2-aminoethyl disulfide)-(4-imidazolecarboxylic acid))-PEG (P(Asp-AED-ICA)-PEG) bearing disulfide bond was designed to respond to biological trigger, glutathione (GSH) 70. These mRNA polyplexes were prepared by adding an appropriate amount of polymer into phosphate-buffered saline (PBS) solution containing mRNA under mixing conditions. The polyplexes could effectively escape from endosomes *via* the proton sponge effect due to the presence of imidazole groups. Once the polyplexes enter the cytoplasm and the disulfide bonds were cleaved by GSH, the cationic polymer would convert into a neutral polymer and further promote the release of mRNA.

### Lipid-polymeric nanoparticles

#### Lipopolyplex (LPP)

Lipid-polymeric nanoparticles (lipopolyplexes, LPPs) are composed of lipid shells and preformed nucleic acid-polycation complex cores, presenting the complementary properties of polymer nanoparticles and liposomes 71, to promote cellular uptake and facilitate endosomal escape of the payload 72. The earliest study on mRNA-based vaccines delivered by LPPs was reported by Hoerr *et al.*, who demonstrated strong immune responses and long-term protection using mRNA 73. In recent years, several types of LPPs for mRNA delivery have been explored using various polymers, such as PEGylated histidylated polylysine (PEG-HpK), PBAE, dendrimers, poly (disulfide amide) (PDSA), and some other polymers 74-76.

**Figure 6:**
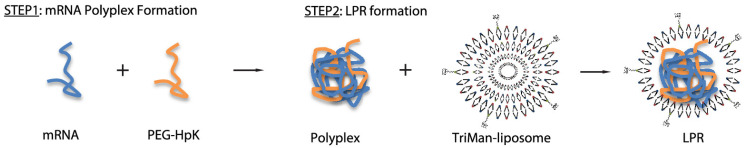
A targeted polymer-lipid hybrid nanoplatform of PEG-HpK and tri-mannosylated liposomes. Adapted with permission from 78, copyright 2018 American Chemical Society.

As reported, LPPs were prepared by adding cationic lipids to the polyplexes composed of mRNA and polymer PEG-HpK. This delivery system could protect mRNA from degradation, facilitate pH-responsive endosome escape, and enhance the protective effect against melanoma tumor 77. In another study, LPPs containing mannose was explored by incubating mRNA/PEG-HpK polyplex with TriManlip100 liposome at an mRNA/liposome ratio of 1:2 (**Figure 6**). These LPPs exhibited favorable DCs targeting ability mediated by the interaction between mannose and its receptors at cell surface, and provided significant tumor-suppression ability 78. A redox-responsive polymer-lipid hybrid system was established by taking advantage of hydrophobic redox-responsive cysteine-based PDSA, G0-C14 and lipid-PEG compounds 79. PDSA was selected to construct a stable core under normal physiological conditions, while achieving a rapid trigger release of payloads in tumor cells with high intracellular glutathione (GSH) concentration. This polymer-lipid hybrid vehicle could deliver p53-encoding mRNA into tumor cells for the expression of tumor suppressor p53 to achieve effective treatment. Remarkably, SW-BIC-213, an mRNA vaccine using LPP as a delivery system, was granted EUA in Laos in 2022, verifying the safety and efficacy of LPP in delivering mRNA.

#### Cationic lipid-assisted nanoparticles (CLANs)

**Figure 7:**
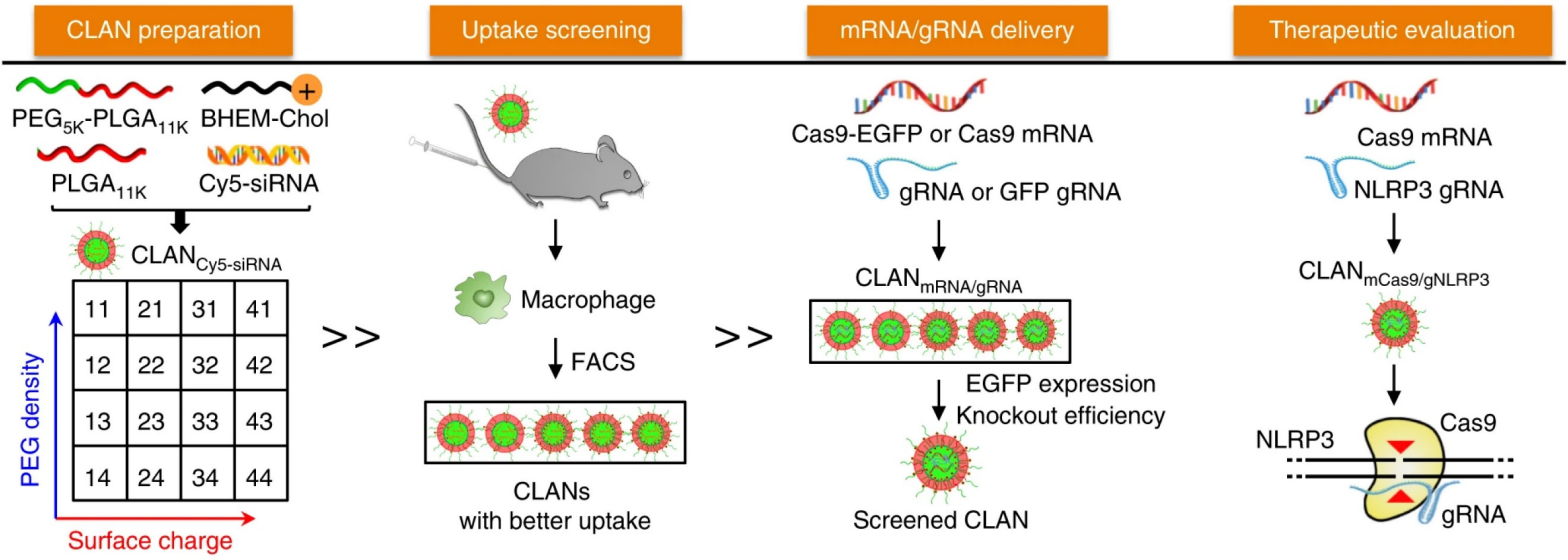
Schematic illustration of the screened CLAN for mCas9/gRNA delivery to macrophages for inflammatory disease treatment. Adapted with permission from 80, copyright 2018 Nature Portfolio.

The CLANs system was first explored by Wang's group in 2009. Major CLANs consisting of PLGA core and cationic lipid shell have been developed by double-emulsion method. PLGA is typically used with cationic lipids to load mRNA via electrostatic interactions due to its uncharged property. For instance, PEG-*b*-PLGA-based nanoparticles assisted by cationic lipid BHEM-Chol was developed to form CLAN nanoparticles, which were then screened for efficiently delivery of Cas9 mRNA and NLRP3 gRNA to macrophages in mice (**Figure 7**) 80. Successful prevention or treatment of sepsis, peritonitis, and type II diabetes in mice was demonstrated by knocking out the NLRP3 gene and suppressing the activation of the NLRP3 inflammasome. Similarly, Zhao *et al.* developed lipid-polymer hybrid nanoparticles by incorporating acid-terminated PLGA into lipid nanoparticles, resulting in prolonged release of mRNA from the nanoparticles and improved mRNA delivery efficiency 81. Overall, CLANs have been proved to be a promising platform for mRNA vaccine delivery.

### Inorganic nanoparticles

Inorganic nanoparticles have been extensively researched for mRNA delivery due to their unique properties, narrow size distribution, and surface chemistry amenable to ligand conjugation 82, 83. Iron oxide and gold nanoparticles are commonly considered as non-toxic nanomaterials 84, but further surface modification is still necessary for better biocompatibility.

**Figure 8:**
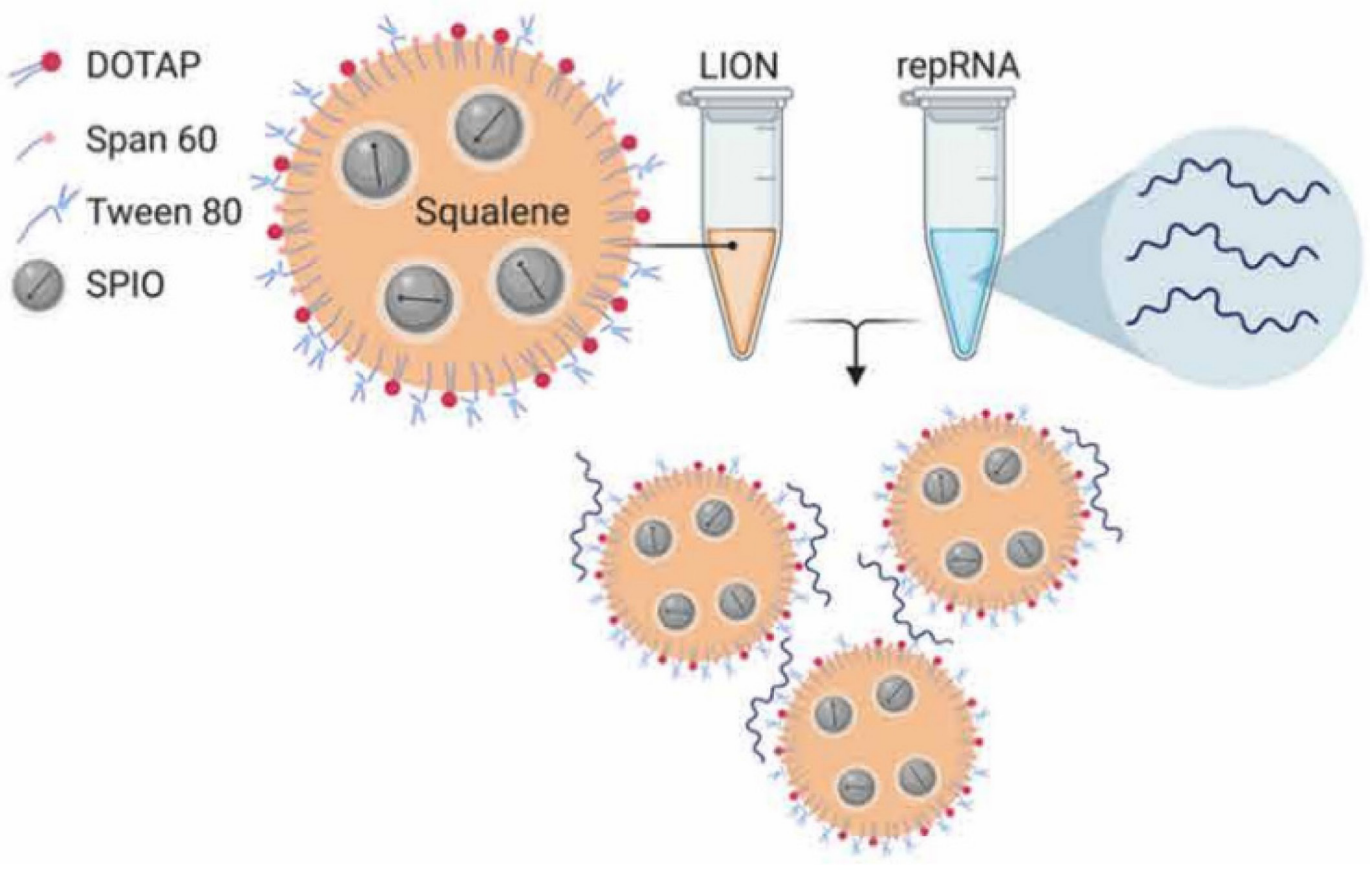
A graphical representation of LION and formation of the vaccine complex after mixing with repRNA. Adapted with permission from 85, copyright 2020 Amer Assoc Advancement Science.

Erasmus *et al.* 85 applied lipid inorganic nanoparticles (LIONs) to deliver replicon RNA (repRNA) encoding SARS-CoV-2 S protein to enhance vaccine stability, delivery, and immunogenicity. LION is a highly stable cationic emulsion composed of squalene, DOTAP, Span 60 and Tween 80, with superparamagnetic iron oxide nanoparticles (SPIO) embedded in the hydrophobic oil phase. When LION was simply mixed with RNA molecules at a 1:1 (v/v) ratio, electrostatic association between anionic repRNA and cationic DOTAP on the surface of LION promoted immediate complex formation, and the 90-nm LION/repRNA-CoV2S nanoparticle delivery system was obtained (**Figure 8**). The nanoparticle vaccine could elicit robust production of anti-SARS-CoV-2 S protein IgG antibody in mice after a single intramuscular injection, and potent T cell responses were induced after a prime/boost regimen. The efficacy was also demonstrated in nonhuman primates, which emphasized the promising prospect of this RNA delivery system. Interestingly, the author claimed that the two-vial approach, where one vial contains the LION formulation and the other vial contains the repRNA vaccine, will be able to provide a significant manufacturing and distribution advantage over formulations requiring complex processes to encapsulate RNA into lipid nanoparticles. The repRNA vaccine and LION formulation can be scaled up and stockpiled separately and then combined on site before use.

Gold nanoparticles (AuNPs) have been used as biomacromolecule carriers in various applications. Mbatha *et al.* 86 aimed to formulate folic-acid-(FA)-modified, poly-amidoamine-generation-5 (PAMAM G5D)-grafted AuNPs (Au:G5D:FA) to deliver mRNA encoding luciferase. Briefly, PAMAM G5D was conjugated to FA, and then the synthesized G5D:FA was further covalently grafted to AuNPs to give rise to Au:G5D:FA, which was subsequently mixed with mRNA at a 4:1 ratio (w/w) to obtain mRNA-loaded nanocomplex. The stable nanocomplex provided excellent protection to the mRNA against RNases. More importantly, the superior *in vitro* transfection efficiency of the nanocomplex indicated the synergistic roles played by both dendrimer and AuNPs in the formulation. The proof-of-concept study has shown potential application of AuNPs in mRNA delivery. However, despite the promising results, most research using inorganic nanoparticles for mRNA delivery is still in the proof-of-concept stage 87. Further formulation and process optimization, as well as more studies in primates and humans, are still necessary for inorganic nanoparticles to broaden their potential applications.

### Cationic nanoemulsion

Cationic nanoemulsions (CNEs) have more recently been described as an mRNA delivery system, mainly due to their safety, well-established production technique, and ability to overcome some major problems associated with liposomes, such as the prevention of particle aggregation in biological fluids 88. CNEs are dispersions of an oil phase in an aqueous phase, generally stabilized by a single cationic lipid or a mixture of phospholipids, non-ionic surfactants, and/or PEG-lipids. The cationic lipid in the formulation plays a crucial role in the complexation of mRNA through electrostatic interactions.

Brito *et al.* 89 described a CNE delivery system to deliver a self-amplifying mRNA vaccine. The CNE is based on Novartis's proprietary adjuvant MF59, which was prepared by mixing an aqueous phase containing Tween 80 with an oil phase containing Span 85, DOTAP, and squalene, followed by homogenization with a homogenizer and microfluidizer. mRNA was added to the CNE at a 7:1 N/P ratio and allowed to complex on ice. The Z-average diameter of the obtained complex was 129 nm with a PDI of 0.117. The protective effect of CNE on mRNA stability was demonstrated by RNase treatment. The obtained vaccine elicited potent immune responses in mice, rats, rabbits, and nonhuman primates, which offers an optimistic prospect for this technology. In addition, Luisi *et al.* 90 developed ZIKV vaccine candidates using self-amplifying mRNA (SAM) delivered by CNE that allows bedside mixing by trained medical personnel. After intramuscular injection of the vaccine candidate, a potent neutralizing antibody response to ZIKV was elicited both in mice and nonhuman primates, and these animals were protected from ZIKV challenge. This study provides a preclinical proof of concept that a SAM-CNE vaccine beside mixing would be particularly useful for a rapid response against a pandemic outbreak. Similarly, researchers developed mRNA vaccines delivered by CNE to fight against human immunodeficiency virus and Venezuelan equine encephalitis virus, both inducing robust protective immunogenicity 91, 92.

While human data on CNE are still pending, it has demonstrated efficacy in multiple preclinical models, indicating significant potential in human clinical evaluation. Further studies are needed to evaluate the safety and efficacy of these delivery systems in humans.

### Others

In addition to nanoparticles discussed above, other mRNA drug delivery systems have been explored.

Cationic peptides or proteins can electrostatically interact with negatively charged mRNA to form nanocomplexes. For example, protamine has been used to form complexes with mRNA and has shown improved transfection efficiency compared to naked mRNA 73. However, the development of this delivery system is challenging due to severe adverse effects 93. Another class of potential mRNA drug delivery systems is cell-penetrating peptides (CPPs), which are mostly cationic peptides of 8-30 amino acids in length. CPPs can spontaneously complex with anionic mRNA through electrostatic interactions upon physical mixing 94-96. Some CPP-mRNA nanocomplexes have yielded enhanced vaccination effects compared to standard liposomal mRNA formulations 97, indicating great potential for the development of mRNA vaccines and therapeutics 98.

Cell-secreted extracellular vesicles, also known as exosomes, are also promising carriers for mRNA delivery due to their favorable biocompatibility, pharmacokinetic properties, and ability to penetrate physiological barriers. However, obtaining adequate exosomes to deliver mRNA has proven challenging, as only a limited number of cells have been reported to secrete sufficient exosomes 99. While post-insertion of short RNA into exosomes *via* electroporation has demonstrated promising therapeutic efficacy 100, challenges remain for delivering large nucleic acids to nano-sized exosomes. Recently, Yang *et al.* 101 developed a cellular nanoporation biochip to stimulate cells to secrete exosomes containing therapeutic mRNA. In glioma mouse models, mRNA-containing exosomes were able to restore tumor-suppressor function, enhance inhibition of tumor growth, and increase survival time, supporting the translational potential of therapeutic exosomes. For exosome-based therapeutics, effective release of exosomes by cells, exosome isolation techniques, and efficient mRNA loading must be prioritized to achieve successful applications 102.

Nano-hydrogel is another promising carrier for mRNA delivery. Fu *et al.* 103 developed a nano-hydrogel system composed of DNA for mRNA delivery, which has superior biocompatibility compared to chemical vehicles. The X-shaped DNA scaffold contains 3 sticky ends that can be paired with the tail and cap regions of the mRNA through a pH-responsive i-motif linker. In this way, the compact nanosphere can be formed under neutral conditions and disintegrate under acidic environments due to the de-hybridization of the i-motif with the scaffold. The nano-hydrogel maintains its nanostructure in physiological conditions and disintegrates upon entering the acidic lysosome of cells *via* endocytosis to release mRNA and facilitate protein expression. Compared to commercial liposomes, the nano-hydrogel exhibits much better biocompatibility with comparable mRNA expression efficiency.

As further interdisciplinary studies are conducted, additional forms of nanocarriers will likely emerge and be applied in the delivery of mRNA, concomitantly addressing some of the existing key challenges.

## Route of administration

The delivery route of mRNA vaccines is crucial in determining their efficacy, as it depends on the anatomical and physiological characteristics of the vaccination sites, such as skin, lymphatic organs, or muscles. These vaccines can be administered either systemically or locally to achieve optimal results.

Systemic administration, either *via* intravenous (i.v.) or intraperitoneal (i.p.) injection, can deliver mRNA drugs directly or indirectly into systemic circulation. Intravenous injection can provide the largest administration volume of mRNA nanoparticles 104, which accumulate in different tissues according to their intrinsic characteristic. After i.v. injection, plain LNPs are absorbed by apolipoprotein E (ApoE) and preferentially target the liver due to the abundant distribution of ApoE receptors. The liver-targeting property of nanoparticles provides more therapeutic potential for protein replacement therapy because the liver is considered a protein factory. Furthermore, specific modifications can be made on mRNA-loaded nanoparticles to target different tissues after i.v. injection, such as the lungs 74, 105, 106, brain 107, and circulating cells 108. However, intravenous administration has some disadvantages. The nanoparticles may be affected by plasma proteins, enzymes, and mechanical forces in the bloodstream, and the delivery vehicles themselves may introduce systemic toxicity 109, 110. Intraperitoneal (i.p.) injection-based mRNA vaccines are administrated inside the peritoneum but outside the serosa of the gastrointestinal tract. These sites exhibit a strong absorptive capacity due to the large peritoneal area, dense blood vessels, and lax lymphatic vessels. In a recent study, liposome-protamine-mRNA nanoparticles demonstrated a good safety profile and delivery capacity of mRNA as well as strong anticancer ability through i.p. injection in a C26 colon cancer model 111.

**Figure 9:**
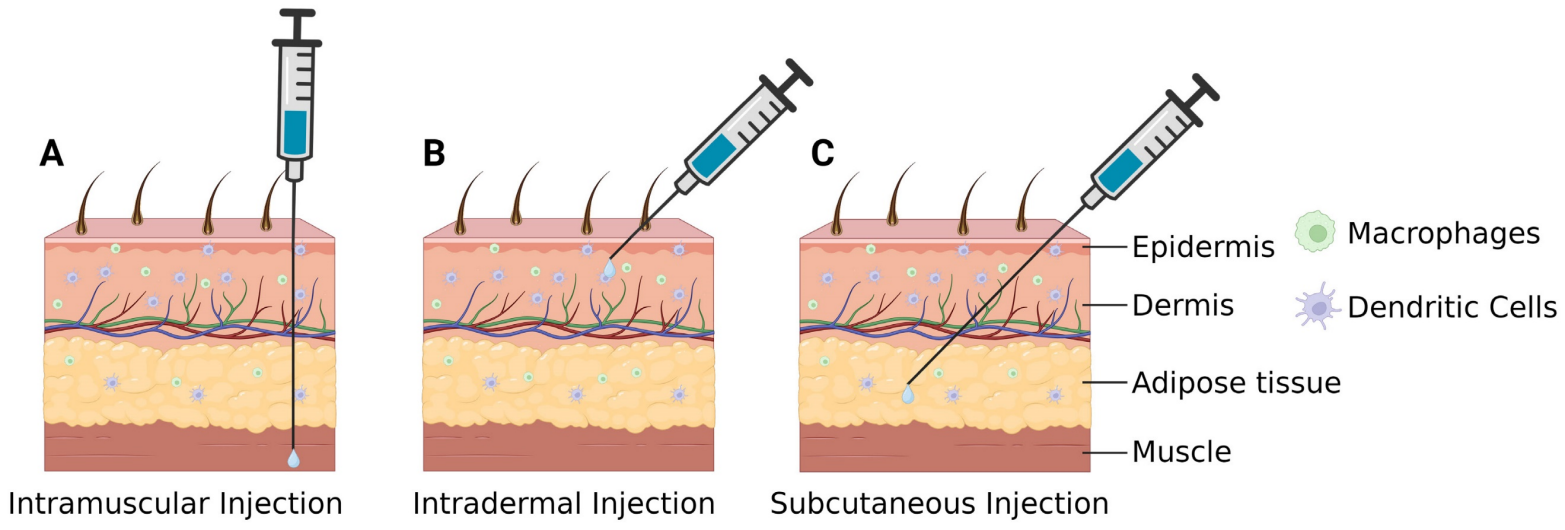
Local administration routes for mRNA vaccines delivery. Created by BioRender.com. (A) Intramuscular injection is the most common mode of vaccination and can deliver mRNA into muscle tissue. (B) Intradermal injection can deliver mRNA into areas with abundant APCs and induce a Th1-type immune response. (C) Subcutaneous injection can deliver mRNA into the loose adipose tissue beneath the skin, allowing for a large injection volume.

Local administration is a delivery method that can decrease the risk of systemic toxicity associated with mRNA and nanoparticle vehicles. Intramuscular (i.m.) injection (**Figure 9A**) is the most commonly used mode of administration for vaccination and can deliver mRNA into muscle tissue 112. This injection method is advantageous for achieving high-dose administration but has specific requirements for particle size and charge; larger particle sizes and charges can hinder delivery and impact efficacy. Currently, all commercially available COVID-19 mRNA vaccines are administered intramuscularly 113. Intradermal (i.d.) injection (**Figure 9B**) can directly deliver the nanoparticles between the epidermis and dermis dermis and can induce a Th1-type immune response effectively due to the high presence of antigen-presenting cells such as Langerhans cells and DCs 114. Therefore, i.d. administration may reduce vaccine dose (dose sparing), thereby reducing costs (including transportation and storage) and expanding the supply chain 115. However, i.d. injection is limited by its low injection volume and increased risk of local adverse effect such as swelling, pain, erythema, and pruritus 116. Subcutaneous (s.c.) injection (**Figure 9C**) involves administrating the mRNA vaccine to the subcutaneous tissue area under the epidermis and dermis. In contrast to the dermis, this layer of skin is primarily composed of a loose network of adipose tissue with few immune cells 117, which allows for a larger injection volume compared to i.d. injections. Notably, the absorption rate is slower in the s.c. region, which may result in mRNA vaccine degradation 118.

Aside from the conventional delivery routes mentioned earlier, other delivery methods such as intranasal instillation, aerosol inhalation, intraocular injection, and intracerebroventricular injection are currently under exploration. Administering mRNA vaccines *via* intranasal instillation is possible because APCs present in the lymph nodes at mucosal sites can efficiently uptake the lipid nanoparticle-mRNA formulations 119. This method can prompt humoral and cell-mediated immune responses 120, 121, and can also deliver mRNA specifically to the lung or brain, offering alternative routes for targeted delivery 122, 123. In principle, aerosol inhalation of mRNA vaccines allows for the targeting of all respiratory tract regions, including the pulmonary region 124, thereby inducing localized immune responses at the pathogen's port of entry 125. However, nebulized mRNA-LNP faces several unique challenges, especially the sensitivity to the shear stress during nebulization which resulted particle structure disintegration and aggregation. To address this kind of problems, Jiang *et al.* 126 altered the nebulization buffer from PBS to 100 mM citrate buffer to increase the LNP charge, and added branched polymeric excipients bPEG20K, to alleviate the nebulization-induced aggregation. The combinatorial strategy yields a significant improvement in lung mRNA delivery. Zhang *et al.* 127 utilized vibrating mesh nebulizers to aerosolize mRNA-LNP formulations and found that the target protein was predominately expressed in the mouse lung following nebulization. Several LNP formulations administered *via* inhalation under clinical studies, such as MRT5005 (NCT03375047), an mRNA-LNP-based therapeutic against cystic fibrosis in phase I/II clinical stage, demonstrate the potential for aerosol inhalation as a delivery route. The feasibility of intraocular and intracerebroventricular injections as administration routes is still under early investigation 128, 129, as they have fewer application scenarios and are much challenging operationally 130.

## Clinical progress of mRNA vaccines for different indications

### Infectious Diseases

Infectious diseases pose a significant threat to human health. To address this issue, mRNA vaccines have emerged as a new technology with the potential to combat infectious diseases effectively. mRNA vaccines offer several advantages, such as ease of manufacturing, acceptable immunogenicity, and a good safety profile, which make them an attractive option for combating infectious diseases 131. The approval of the COVID-19 vaccines BNT162b2 and mRNA-1273 by the US Food and Drug Administration (FDA) marked a significant milestone for mRNA technology (**Table 3**). mRNA is now recognized as a promising vaccine model for infectious diseases, and more research is needed to validate the efficacy of mRNA.

#### SARS-CoV-2

The global impact of the COVID-19 pandemic is staggering, with over 772 million people infected and more than 6.9 million fatalities until 22 November 2023. Among the effective vaccine candidates against the virus are Pfizer-BioNTech's BNT162b2 and Moderna's mRNA-1273, both authorized by the FDA in 2020. BNT162b2 is made up of ionizable lipid ALC-0315 and nucleoside-modified mRNA. In a phase III trial with over 43,000 participants, the vaccine demonstrated a remarkable 95% overall efficacy in preventing COVID-19 infection and was also effective in a mass vaccination campaign 132, 133.

Moderna's mRNA-1273 vaccine utilized ionizable lipid SM-102 to encapsulate modified mRNA in lipid nanoparticles. In addition to mRNA-1273, thermostable vaccine candidates are also being developed, including ARCoV, a collaboration between the Academy of Military Sciences of China and Abogen Biosciences. ARCoV has been shown to remain stable for a week at 25°C, making it ideal for distribution in regions with limited access to refrigeration. In a phase I clinical trial with multiple dosing groups, ARCoV was found to be safe and well-tolerated among all participants. Furthermore, the vaccine was capable of eliciting robust humoral and cellular immune responses (ChiCTR2000039212) 134. Several other promising self-amplifying mRNA vaccine candidates are also under development by Arcturus Therapeutics. Arcturus Therapeutics is also actively involved in the development of several promising self-amplifying mRNA vaccine candidates, which have the potential to optimize immune responses and reduce the required vaccine doses. These vaccine candidates are currently in various stages of preclinical and clinical development, and show promising results in animal models and early-phase clinical trials. Arcturus Therapeutics is dedicated to advancing these innovative vaccine candidates to combat infectious diseases, including the ongoing COVID-19 pandemic.

#### Influenza viruses

The influenza virus is a widespread human respiratory pathogen that presents a major challenge to public health and the global economy. Its ability to undergo antigenic drift and antigenic transfer allows for the emergence of new influenza strains, resulting in regular seasonal epidemics and occasional pandemics. These biological phenomena drive the need for continuous vaccine updates to provide adequate protection against evolving viral strains 135. The ongoing emergence of new strains of influenza virus underscores the urgent need for effective vaccines. mRNA-based vaccines are one promising avenue for improving influenza prevention. Multiple studies have shown that mRNA-based influenza vaccines can trigger a potent and durable immune response against the virus 136. For example, two phase I clinical trials of Moderna's mRNA-LNP vaccines for pandemic avian influenza H10N8 and H7N9 influenza viruses (NCT03076385 and NCT03345043) demonstrated the safety and immunogenicity of the vaccine in healthy adults. The trials showed that the mRNA-LNP vaccine generated a strong humoral immune response against the targeted influenza viruses in the absence of adjuvants. These results indicate that the use of LNP-formulated mRNA is a promising vaccine platform for influenza prevention, and the ongoing development of mRNA vaccines holds great potential to further improve influenza prevention and control 137.

#### Rabies virus

Rabies is a life-threatening neurological disease that can affect various warm-blooded animals, including humans. CureVac has developed the mRNA vaccine candidate, CV7201, for rabies using the cationic polypeptide protamine as a delivery carrier. Preclinical studies of CV7201 in mice and pigs have indicated that it can elicit effective humoral and T-cell immune responses 138. A phase I clinical trial of CV7201 (NCT02241135) demonstrated that the vaccine had a favorable safety profile and induced immunogenicity against rabies virus at low doses 93. However, CV7201 had a higher neutralizing antibody when injected with a needle-free device compared with a needle-syringe, which indicated that the efficacy of CV7201 appeared to be largely dependent on the vaccination method and would thus limit the application of this vaccine 93. CureVac has since developed an alternative LNP-based delivery system, CV7202 (NCT03713086), which has shown improved results in clinical trials. The low-dose mRNA-LNP vaccine was well-tolerated and safe, with all participants demonstrating valid titers above 0.5 IU/mL 139. Comparison of clinical trial data between CV7201 and CV7202 showed that the LNP-based delivery system was superior to the protamine system in inducing an effective immune response against rabies virus. These findings highlight the importance of the mRNA delivery system in the development of successful mRNA vaccines.

#### Human immunodeficiency virus (HIV)

HIV is a global health challenge that weakens the immune system by destroying immune cells, making individuals more vulnerable to various infections and diseases. There were approximately 38.4 million people across the globe with HIV in 2021 according to the Global Statistics. Despite years of research, effective preventive vaccines against HIV are still urgently needed. However, many HIV vaccine candidates have failed in trials. The Sanofi and GSK HIV vaccine, once considered the most promising candidate, was declared a failure in 2020 140. Johnson & Johnson's Imbokodo vaccine demonstrated a protective efficiency of only 25.2%, leading to a halt in further development. Moderna's experimental mRNA HIV vaccine, developed using the same platform technology as their COVID-19 mRNA vaccine, has shown significant promise in mice and non-human primates. The vaccine was found to be safe and capable of inducing the production of neutralizing antibodies and a potent immune response against HIV 141. Currently, a phase I clinical trial (NCT05414786) is underway to evaluate the safety and immunogenicity of the mRNA-1644 vaccine candidate. These positive results suggest that mRNA-based vaccines represent an innovative approach to developing effective HIV vaccines and hold tremendous potential for revolutionizing the field of HIV research.

#### Others

Moderna has developed several LNPs-formulated vaccines, including an mRNA vaccine candidate (mRNA-1893) for the Zika virus currently undergoing a phase II clinical trial (NCT04917861). The vaccine encodes for structural proteins of the Zika virus and has shown promising results in a Phase I study, with both 10 µg and 30 µg dose levels seroconverting a majority of seronegative participants and boosting seropositive participants with good tolerability. Another vaccine in development by Moderna is the cytomegalovirus (CMV) vaccine (mRNA-1647). CMV is a major cause of birth defects worldwide and is particularly concerning for pregnant women 142, who can transmit the infection to their unborn babies. There is currently no approved vaccine for CMV infection.

The mRNA-1647 vaccine is composed of six mRNAs encoding for two CMV surface proteins (the pentamer complex and glycoprotein B) and is currently undergoing Phase III clinical trials (NCT05085366) 143. Moderna is also developing vaccine candidates against other latent viruses, including Epstein-Barr virus (EBV), herpes simplex virus (HSV), and varicella-zoster virus (VZV). These vaccine candidates, like Moderna's other mRNA vaccines, utilize LNP formulations and hold promise as new tools for preventing and controlling viral infections. Continued research and clinical trials will be essential to assess their safety, efficacy, and potential impact on public health (**Table 3**).

### Cancer

Cancer vaccines offer a promising alternative to cancer immunotherapy by providing preventive and therapeutic effects. By targeting tumor-associated antigens (TAAs) or tumor-specific antigens (TSAs), vaccines can specifically attack and destroy malignant cells overexpressing these antigens while activating immune memory to achieve long-lasting therapeutic effects. Therefore, mRNA vaccines are expected to be an effective means of cancer treatment.

TAAs are highly expressed on the surface of tumor cells and can be recognized by the immune system, thus achieving a killing effect. As a result, many mRNA cancer vaccines based on TAAs are under development, with BioNTech's BNT111 showing the fastest progress, currently in phase I and phase II clinical trials (NCT02410733). This vaccine is formulated with LPX as a vehicle and mRNAs that encode a fixed set of four melanoma-associated antigens (New York oesophageal squamous cell carcinoma 1 (NY-ESO-1), melanoma-associated antigen A3 (MAGE-A3), tyrosinase, and transmembrane phosphatase with tensin homology (TPTE)). The phase I clinical trials showed BNT111 to be well tolerated in patients with advanced melanoma and capable of mediating a long-lasting immune response, linked to the activation and strong expansion of tumor antigen-specific CD4^+^ and CD8^+^ T cells 144. BioNTech also has other mRNA vaccines targeting TAAs, such as BNT112 for prostate cancer (encoding a fixed set of five prostate cancer-associated antigens), BNT113 (encoding two oncoproteins, E6 and E7), and BNT116 (encoding a fixed set of antigens frequently expressed in non-small cell lung cancer (NSCLC)). In contrast to BioNTech, previous investigations into mRNA cancer vaccines at CureVac used protamine as a delivery vehicle, which had limited protein expression level speculated to be caused by excess protamine and mRNA binding. Since then, the pipeline appears to have been terminated. At present, CureVac's new pipeline CV8102 uses cationic peptide as a carrier and is currently in phase I clinical study for cancer treatment (NCT03291002) 145.

The genetic instability of tumor cells often results in a large number of mutations. The expression of non-synonymous mutations can lead to the production of tumor-specific antigens (TSAs), known as neoantigens. Some neoantigens can be expressed, processed, and presented on the cell surface, where they can be recognized by T cells. These neoantigens are unique to each patient and have become ideal targets for cancer immunotherapy. BioNTech has developed several clinical neoantigen vaccine candidates for the treatment of cancer. BNT122 (RO7198457) is an individualized cancer vaccine that encodes up to 20 patient-specific tumor neoantigens. Its aim is to induce a potent immune response against the patient's unique tumor. The vaccine is currently being evaluated in a phase II trial. Recently, Moderna and Merck have announced the phase 3 trial of their personalized cancer vaccine, mRNA-4157 (also known as V940), which is formulated with up to 34 neoantigens and administered intramuscularly using lipid nanoparticles (LNPs). The trial, launched on 26 July 2023, will test the efficacy of mRNA-4157 combined with pembrolizumab (Keytruda) as a combination therapy for melanoma (NCT05933577). Stemirna Therapeutics has also developed an mRNA personalized cancer vaccine that uses encapsulated mRNA encoding tumor neoantigens in LPP. The vaccine is currently undergoing clinical phase I trial (NCT05198752).

In addition, mRNA-LNPs can be used to relieve the tumor immunosuppressive microenvironment by delivering cytokines or co-stimulatory molecules, producing anti-tumor effects. This approach has been utilized in mRNA-2752, an LNP-encapsulated mRNA vaccine encoding T cell co-stimulator (OX40 Ligand, OX40L) and pro-inflammatory cytokines (IL-23 and IL-36γ). The vaccine is currently in phase I clinical trials (NCT03739931), in which mRNA-2752 is administered intratumorally every 2 weeks for up to 7 doses as monotherapy or in combination with immune checkpoint inhibitor (ICI) durvalumab 146. Results showed that mRNA-2752 was well tolerated and of the 17 patients evaluated, 1 had a partial response, 6 had stable disease, and 10 had progressive disease. Similarly, BNT131 (SAR441000), encoding IL-12sc, IL-15sushi, IFN-α, and granulocyte-macrophage colony-stimulating factor (GM-CSF), is also being investigated (NCT03871348).

The rapid development of delivery systems and neoantigens has shown great potential in clinical trials for the application of mRNA therapy in cancer, with the possibility of improving cancer immunotherapies in the tumor microenvironment (TME) by nanoparticle-encapsulated mRNAs (**Table 4**).

### *In Vivo* chimeric antigen receptor (CAR) T

The approval of CAR-T cell therapy by the FDA in 2017 marked the beginning of a new era in cell therapy. However, CAR-T cell therapy requires the collection of the patient's own T cells, followed by genetic reprogramming to enable them to recognize and kill cancer cells. The entire process is complex, time-consuming, and expensive. To overcome these challenges, a potential solution is to engineer T cells *in vivo* using a delivery system, especially LNPs, to deliver mRNA encoding CAR.

The team of Michael J. Mitchell constructed an ionizable library for the delivery of mRNA to primary human T cells. The results showed that CD19-coding mRNA could be successfully delivered to primary T cells, producing functional CAR T cells 147. Furthermore, Rurik *et al.* successfully generated CAR-T cells *in vivo* and created an antifibrotic effect in murine heart tissue using mRNA-LNPs. LNPs modified with CD5 targeted T cells and delivered mRNA for CAR that enabled them to attack activated fibroblasts and reduce cardiac fibrosis 148. While CAR-T based on mRNA transduction may not provide long-lasting CAR-T cells suitable for cancer treatment, it is safer for myocardial fibrosis. The development of *in vivo* generated CAR-T cell therapy with only one injection of mRNA-LNPs holds great promise to overcome the current problems of complex processes, long treatment cycles, and high prices associated with CAR-T therapy.

### Others

mRNA-based protein replacement therapy has shown promise in treating fibrosis. Patients with fibrosis often suffer from recurrent airway infections and chronic respiratory problems due to defective fibrosis transmembrane conductance regulator (CFTR), the chloride channel on epithelial cells. LNPs encapsulating CFTR-coding mRNA can restore chloride secretion in CFTR knockout mice.

Translate Bio has initiated a clinical trial (NCT03375047) to evaluate the safety and tolerability of inhalable mRNA-LNPs formulation (MRT5005) in patients with fibrosis. In this study, patients received a single dose of MRT5005 at three different dose levels, and the results showed that MRT5005 was well tolerated with improved lung function in the medium-dose group.

Protein replacement therapy using mRNA-LNP formulations can also treat inherited metabolic diseases, such as propionic acidemia (PA) (NCT04159103). PA is a rare metabolic disorder caused by the deficiency of the PCC enzyme, with no approved therapies. Moderna's mRNA therapy for PA (mRNA-3927) is a combination therapy encoding for PCCA and PCCB to form an active PCC enzyme (**Table 5**).

Gene editing systems provide the opportunity to correct mutated genes, especially in genetic diseases. Intellia Therapeutics has initiated a phase I clinical trial (NCT04601051) to investigate the safety, kinetics, and pharmacodynamics of NTLA-2001 (LNPs encapsulating CRISPR-Cas9) in patients with hereditary transthyretin amyloidosis (ATTR). ATTR is a progressively fatal disease characterized by the accumulation of amyloid fibrils composed of misfolded transthyretin (TTR) protein in tissues. NTLA-2001 aims to edit TTR in hepatocytes, leading to a decrease in the production of both wild-type and mutant TTR after a single administration 149. Updated data from phase I over 60 patients showed consistent, deep and durable serum TTR reduction, which means that gene editing therapies may make breakthrough progress in the future.

## Market trend

The outbreak of COVID-19 has accelerated the development of mRNA technology platforms, leading to the creation of over 200 products as of 2023. These products rely on delivery systems such as LNP, LPX, LPP, LUNAR^®^, and other nanoparticles, although LNP remains the most common formulation. Initially focused on prophylactic vaccines, the number of mRNA products is gradually shifting towards therapeutic vaccines, with oncology being a particularly notable area of progress. mRNA-based therapies show great potential, and there are currently approximately 150 clinical trials for mRNA-based cancer treatments listed on ClinicalTrials.gov.

BioNTech, Moderna, and CureVac have robust mRNA-based pipelines, with many companies developing mRNA vaccines for cancer therapy, including TAA vaccines, personalized cancer vaccines (PCV), and cytokines, alongside anti-infection prophylactic mRNA vaccines. BioNTech and Moderna's clinical phase II products lead the way, with few emerging mRNA enterprises entering the clinical stage. In the realm of prophylactic vaccines, Abogen's COVID-19 vaccine has entered clinical phase III, while Stemirna, Lifanda Bio, CanSino, and CSPC have also made significant progress. However, other products are still in preclinical stages and lagging behind the international market leaders.

An increasing number of companies are collaborating with each other to develop combination therapies, such as combining mRNA vaccines with PD-1 inhibitors or cell therapy. Additionally, Capstan Therapeutics is poised to advance *in vivo* CAR-T therapy to the clinical stage using LNP and mRNA technology, which target two types of cells: T cells for treating hematological cancers, solid tumors, fibrotic diseases, and autoimmune diseases, and hematopoietic stem cells for treating inherited blood disorders.

According to Xie *et al.*'s analysis and prediction, the global mRNA technology market was mainly driven by COVID-19 in 2021. Pfizer and BioNTech's COVID-19 mRNA vaccine generated nearly $40.4 billion in revenue, while Moderna saw a 2000% increase in annual revenue due to the same. However, with COVID-19 being controlled and the population receiving universal vaccination, the market size is expected to decrease. From 2025 to 2035, mRNA products will be introduced successively, with prophylactic vaccines remaining the mainstream products. With the development of therapeutic vaccines and other mRNA products, the market is expected to reach approximately $23 billion by 2035 150.

## Conclusions and prospective

mRNA-based therapy holds great promise for treating infectious diseases, cancer, and inheritable diseases. The rapid development of biomaterials and nanotechnology could greatly help solve various problems such as delivery issues, limited stability, cell targeting and transfection efficiency. Non-viral vectors, such as LNPs, can effectively load mRNA, improve transfection efficiency, trigger immune responses, and enhance antibody titers. This has been exemplified by mRNA vaccines for COVID-19. Further development and clinical applications of mRNA-based therapeutics can be facilitated by the use of LPX, polyplexes, LPPs, and others. However, the safety, effectiveness, and functionality of these delivery vectors need to be explored. For lipid nanoparticles, biodegradable lipid-based nanoparticles can be quickly eliminated from plasma and tissues, enhancing their safety and tolerability. Cationic lipids determine the delivery efficiency and transfection efficiency of lipid nanoparticle-mRNA formulations, and toxicity of vectors is related to cationic lipid structure. Ionizable lipids endow vectors with low toxicity stealth ability. Regulating head groups and hydrophobic tails of lipids can enhance the delivery efficiency of mRNA by increasing cellular uptake and endosomal escape of mRNA-loaded lipid nanoparticles. Adjusting lipid structures can also achieve organ-specific and cell-specific delivery of lipid nanoparticles. In the face of these challenges, naturally derived membrane lipids (such as exosomes and cell membranes) may provide another option for mRNA delivery.

For other delivery vectors, polymeric nanoparticles exhibited diversified delivery strategies owing to the versatility, tunability and scalability of polymers. Both targeted functional groups modified polymers and responsive polymers can effectively delivery mRNA *in vitro* and *in vivo*. However, as clinical vaccine candidates, polymeric nanoparticle-mRNA formulations require comprehensive safety evaluation of polymers and their degradation products, as well as reliable quality controls based on the complexity of polymer structures before entering the market. Hybrid nanoparticles, such as LPPs or CLANs, may improve mRNA delivery potency by integrating the advantages of individual components, whereas the biodegradability of components and their degradation products should be evaluated.

In recent years, numerous preclinical and clinical trials of mRNA vaccines for various diseases have also confirmed the reliability of these mRNA-based treatment nanoplatforms. mRNA vaccines on the market are primarily administered by injection, creating opportunities for the development of new delivery technologies and dosage forms. However, due to the limitations of mRNA, additional efforts are needed to explore novel nanoplatforms and optimized formulations that can achieve effective *in vivo* delivery, minimal toxicity, and favorable potency. Therefore, ongoing research efforts are focused on creating better mRNA delivery vehicles with enhanced performance and safety for treating a range of diseases.

## Figures and Tables

**Table 1:** Commercial COVID-19 mRNA-LNP vaccines (Partial).

Category	Moderna	BioNTech/Pfizer
Product	mRNA-1273	BNT162b2
mRNA dose	100 μg	30 μg
Ionizable cationic lipid	SM-102	ALC-0315
Phospholipid	DSPC	DSPC
Cholesterol	Cholesterol	Cholesterol
PEG-lipid	DMG-PEG2000	ALC-0159
Molar ratios	50:10:38.5:1.5	46.3:9.4:42.7:1.6
Molar N/P ratios	6	6
Other excipients	Potassium chloride	Sodium acetate
	Sodium chloride	Sucrose
	Sucrose	Water for injection
	Water for injection	/

Note: SM-102, heptadecan-9-yl 8-((2-hydroxyethyl)(6-oxo-6-(undecyloxy)hexyl)amino)octanoate; ALC-0315, (4-hydroxybutyl)azanediyl bis(hexane-6,1-diyl)bis(2-hexyldecanoate); ALC-0159, (2-hexyldecanoate), 2-[(polyethylene glycol)-2000]-N,N-ditetradecylacetamide; DSPC, 1,2-distearoyl-*sn*-glycero-3-phosphocholine; DMG-PEG 2000, 1,2-dimyristoyl-rac-glycero-3-methoxypolyethylene glycol-2000.

**Table 2:** Selected polymer-based vehicles for mRNA delivery

Category	Polymers	mRNA delivered	*In vitro* models	Route of administration	In vivo models	Category
Polyethyleneimine (PEI)	PEI_2k_-stearic acid (PSA)	HIV-1 gag	DC2.4	s.c.	BALB/c mice	Polyethyleneimine (PEI)
	CD-PEI	HIV gp120, OVA, Luc	DC2.4, MDCK, Calu-3	intranasal administration	BALB/c mice	55, 56
	PEI-*g*-PEG	Luc	PC3, DC2.4	i.v.	BALB/c mice	57
Polyester	PACE	FLuc	Expi293F	i.v.	BALB/c mice	Polyester
	PBAE-*co*-PCL	FLuc	Hela	i.v.	C57BL/6 mice	62
	PBAE-PGA-Ab	eGFP	K562-CD19, Jurkat-E6	i.v.	NOD.Cg-*Prkdc^scid^ Il2rg^tm1Wjl^*/SzJ (NSG) mice	63
Dendrimers	PAMAM	Luc, cOVA	BHK21	intranasal administration	C57BL/6 and BALB/c mice	Dendrimers
	IAJD	Luc	HEK 293T	retro-orbital sinus injection	BALB/c mice	65
Others	Chitosan coated PLGA	Z3 nec-mRNA	A549, MLE12	i.p.	BALB/c mice, transgenic SP-B mice	Others
	Oligoalkylamines-*g*-PAA_8k_	FLuc	A549, HepG2, NIH 3T3	i.v.	BALB/c mice	67
	P(Asp-AED-ICA)-PEG	GFP	HEK 293, RAW 264.7	/	/	68

Note: i.v., intravenous; i.m., intramuscular injection; s.c., subcutaneous injection; i.p., intraperitoneal injection.

**Table 3:** Summary of on-going clinical studies of mRNA vaccines for infectious disease.

Name of product	Payload (for example, antigen or protein)	Disease	Route of administration	NCT Number	Study phase	Trial status	Sponsor/collaborators	Vehicle
mRNA-1010	Encoding for hemagglutinin (HA) glycoproteins of the four influenza strains	Seasonal Influenza	i.m.	NCT04956575	I/II	Recruiting	Moderna	LNP
mRNA-1010	Encoding for hemagglutinin (HA) glycoproteins of the four influenza strains	Seasonal Influenza	i.m.	NCT05827978	III	Active, not recruiting	Moderna	LNP
mRNA-1020, mRNA-1030	Eight mRNAs targeting both hemagglutinin and neuraminidase	Seasonal Influenza	i.m.	NCT05333289	I/II	Completed	Moderna	LNP
mRNA-1189	Four mRNA's that encode EBV envelope glycoproteins	Epstein-Barr Virus Infection	i.m.	NCT05164094	I	Recruiting	Moderna	LNP
mRNA-1345	Stabilized prefusion F protein	Respiratory syncytial virus	i.m.	NCT05127434	II/III	Active, recruiting	Moderna	LNP
mRNA-1644	eOD-GT8 60mer mRNA	HIV-1-infection	i.m.	NCT05414786	I	Active, recruiting	Moderna	LNP
mRNA-1644v2-Core	Core-g28v2 60mer mRNA	HIV-1-infection	i.m.	NCT05414786	I	Active, recruiting	Moderna	LNP
mRNA-1647	Six mRNAs coding for pentamer viral antigen and gB protein of CMV	CMV infection	i.m.	NCT04232280	II	Completed	Moderna	LNP
mRNA-1647	Six mRNAs coding for pentamer viral antigen and gB protein of CMV	CMV infection	i.m.	NCT05085366	III	Recruiting	Moderna	LNP
mRNA-1653	Two distinct mRNA sequences encoding the fusion proteins of hMPV and PIV3	Human Metapneumovirus and Human Parainfluenza Infection	i.m.	NCT04144348	I	Completed	Moderna	LNP
mRNA-1893	Structural proteins of Zika virus	Zika virus	i.m.	NCT04917861	II	Active, recruiting	Moderna	LNP
mRNA-1283	SARS-CoV-2	SARS-CoV-2	i.m.	NCT05137236	II	Completed	Moderna	LNP
mRNA-1273	Stabilized S protein of SARS-CoV-2	SARS-CoV-2	i.m.	NCT04405076	II	Completed	Moderna	LNP
mRNA-1273	Stabilized S protein of SARS-CoV-2	SARS-CoV-2	i.m.	NCT04470427	III	Completed	Moderna	LNP
mRNA-1273	Stabilized S protein of SARS-CoV-2	SARS-CoV-2	i.m.	NCT04649151	III	Active, not recruiting	Moderna	LNP
mRNA-1273	Stabilized S protein of SARS-CoV-2	SARS-CoV-2	i.m.	NCT04796896	II/III	Active, recruiting	Moderna	LNP
mRNA-1273	Stabilized S protein of SARS-CoV-2	SARS-CoV-2	i.m.	NCT04969276	II	Completed	Moderna	LNP
mRNA-1273	Stabilized S protein of SARS-CoV-2	SARS-CoV-2	i.m.	NCT04811664	III	Completed	NIAID	LNP
mRNA-1273	Stabilized S protein of SARS-CoV-2	SARS-CoV-2	i.m.	NCT04894435	II	Active, recruiting	Canadian Immunization Research Network	LNP
mRNA-1273.211	Combines mRNA-1273 and mRNA-1273.351 in a single vaccine	SARS-CoV-2	i.m.	NCT04889209	I/II	Completed	NIAID	LNP
mRNA-1273.351	Codes for the full-length prefusion stabilized S protein of the SARS-CoV-2 B.1.351 variant	SARS-CoV-2 B.1.351 variant	i.m.	NCT04785144	II	Completed	Moderna	LNP
BNT162 (four types)	Four different SARS-CoV-2 vaccines: BNT-162a1, BNT162b1, BNT162b2, BNT162c2	SARS-CoV-2	i.m.	NCT04380701	I/II	Active, recruiting	BioNTech-Pfizer	LNP
BNT162b2	SARS-CoV-2	SARS-CoV-2	i.m.	NCT04889209	I/II	Completed	NIAID	LNP
BNT162b2	SARS-CoV-2	SARS-CoV-2	i.m.	NCT04969250	III	Completed	NIAID	LNP
BNT162b2	SARS-CoV-2	SARS-CoV-2	i.m.	NCT04816643	II/III	Active, recruiting	BioNTech	LNP
BNT164	Tuberculosis	Tuberculosis	i.m.	NCT05537038	I	Recruiting	BioNTech	Not declared
CVnCoV	SARS-CoV-2	SARS-CoV-2	i.m.	NCT04515147	IIa	Completed	CureVac	LNP
CVnCoV	SARS-CoV-2	SARS-CoV-2	i.m.	NCT04652102	II/III	Completed	CureVac	LNP
CV0501	SARS-CoV-2 Omicron	SARS-CoV-2 Omicron	i.m.	NCT05477186	I	Completed	GSK	LNP
CV7202	RABV-G mRNA	Rabies	i.m.	NCT03713086	I	Completed	CureVac	LNP
CVSQIV	four differentinfluenza strains	seasonal influenza	i.m.	NCT05252338	I	Completed	CureVac/GSK	Not declared
ARCT-154	SARS-CoV-2	SARS-CoV-2	i.m.	NCT05037097	I/II	Unknown	Arcturus Therapeutics	LUNAR®
ARCT-154	SARS-CoV-2	SARS-CoV-2	i.m.	NCT05012943	II/III	Completed	Arcturus Therapeutics	LUNAR®
ARCT-021	SARS-CoV-2	SARS-CoV-2	i.m.	NCT04668339	II	Terminated	Arcturus Therapeutics	LUNAR®
ChulaCov19-BNA159	SARS-CoV-2	SARS-CoV-2	i.m.	NCT05231369	I/II	Recruiting	ChulalongkornUniversity	LNP
MRT5407	Quadrivalent Influenza	Influenza	i.m.	NCT05553301	I/II	Active, not recruiting	Sanofi	LNP
COVID-19 mRNA vaccine	SARS-CoV-2	COVID-19	i.m.	NCT05373485	I	Active, not recruiting	CanSino	LNP
COVID-19 mRNA vaccine	SARS-CoV-2	COVID-19	i.m.	NCT05373472	II	Active, not recruiting	CanSino	LNP
COVID-19 mRNA vaccine	SARS-CoV-2	SARS-CoV-2	i.d.	NCT05315362	II	Recruiting	Leiden UniversityMedical Center	Solid microneedle skin patch
SYS6006	SARS-CoV-2	SARS-CoV-2	i.m.	NCT05439824	II	Active, not recruiting	CSPC ZhongQiPharmaceutical Technology	Not declared
LVRNA009	SARS-CoV-2	SARS-CoV-2	i.m.	NCT05352867	II	Completed	AIM Vaccine	LNP
ABO1009-DP	SARS-CoV-2 Omicron	SARS-CoV-2 Omicron	i.m.	NCT05433194	I	Active, not recruiting	Abogen Biosciences	LNP
iHIVARNA-01	An HIV immunogen to induce T cell responses against relatively conserved, vulnerable portions of the virus, HTI	HIV	i.n.	NCT02888756	II	Terminated	Hivarna consortium, Etherna	LNP

Note: i.m., intramuscular injection; i.n., intranodal injection; i.d., intradermal injection.

**Table 4:** Summary of on-going clinical studies of mRNA vaccines for cancer.

Name of product	Payload (for example, antigen or protein)	Disease	Route of administration	NCT Number	Study phase	Trial status	Sponsor/collaborators	Vehicle
mRNA-4157	Twenty tumor-associated antigens	Melanoma	i.m.	NCT03897881	II	Recruiting	Moderna	LNP
mRNA-4157	Twenty tumor-associated antigens	Melanoma	i.m.	NCT05933577	III	Recruiting	Moderna	LNP
mRNA-4359	IDO and PD-L1	Advanced Solid Tumors	i.m.	NCT05533697	I/II	Recruiting	Moderna	LNP
mRNA-5671(V941)	Target G12D, G12V, G13D or G12C driver mutations in the KRAS gene	NeoplasmsCarcinoma, Non-Small-Cell LungPancreatic NeoplasmsColorectal Neoplasms	i.m.	NCT03948763	I	Completed	Merck Sharp & Dohme LLC	LNP
BNT111	Four melanoma-associated antigens	Melanoma	i.v.	NCT04526899	II	Active, not recruiting	BioNTech	LPX
BNT112	Five prostate cancer-associated antigens	Prostate Cancer	i.v.	NCT04382898	I/II	Active, not recruiting	BioNTech	LPX
mRNA-2752	Encoding OX40L T cell co-stimulator, IL-23 and IL-36γ pro-inflammatory cytokines	Advanced Malignancies	i.t.	NCT03739931	I	Recruiting	Moderna	LNP
MEDI1191	IL-12	Solid Tumors	i.t.	NCT03946800	I	Completed	MedImmune LLC	LNP
BNT113	Two oncoproteins, E6/E7	Unresectable Head and Neck Squamous Cell CarcinomaMetastatic Head and Neck CancerRecurrent Head and Neck Cancer	i.v.	NCT04534205	II	Recruiting	BioNTech	LPX
BNT116	Tumor-linked antigens found to be commonly expressed in NSCLC	NSCLC	i.v.	NCT05557591	II	Recruiting	BioNTech	LPX
BNT122(RO7198457)	Up to 20 patient-specific tumor neoantigens	Colorectal Cancer Stage II/III	i.v.	NCT04486378	II	Recruiting	BioNTech	LPX
BNT142	bispecific antibody, against CLDN6 and CD3	Solid Tumor	i.v.	NCT05262530	I/II	Recruiting	BioNTech	LNP
BNT151	IL-2	Solid Tumor	i.v.	NCT04455620	I/II	Recruiting	BioNTech	LNP
BNT152/BNT153	IL-7/IL-2	Solid Tumor	i.v.	NCT04710043	I	Recruiting	BioNTech	LNP
BNT131(SAR441000)	IL-12sc, IL15-sushi, GM-CSF, IFNα	Metastatic Neoplasm	i.t.	NCT03871348	I	Active, not recruiting	SanofiBioNTech RNA Pharmaceuticals GmbH	Not declared
CV8102	Agonist to TLR-7/-8 and RIG-I	Melanoma (Skin)Squamous Cell Carcinoma of the SkinCarcinoma, Squamous Cell of Head and NeckCarcinoma, Adenoid Cystic	i.t.	NCT03291002	I	Unknown status	CureVacSyneos HealthCromos Pharma LLC	Cationicpeptide
SW1115C3	Neoantigen	Solid Tumor	s.c.	NCT05198752	I	Recruiting	Stemirna Therapeutics	LPP
SW1115C3	Neoantigen	Solid Tumor	s.c.	NCT05949775	Not Applicable	Not yet recruiting	Stemirna Therapeutics	LPP

Note: i.t., intratumoral injection; i.v., intravenous; i.m., intramuscular injection; s.c., subcutaneous injection.

**Table 5:** Summary of on-going clinical studies of mRNA for other applications.

Name of product	Payload (for example, antigen or protein)	Disease	Route of administration	NCT Number	Study phase	Trial status	Sponsor/collaborators	Vehicle
MRT5005	Cystic fibrosis transmembrane conductance regulator	Cystic fibrosis	Inhalation	NCT03375047	I/II	Unknown status	Translate Bio	LNP
LUNAR-OTC (ARCT-810)	Ornithine transcarbamylase	Ornithine transcarbamylase deficiency	i.v.	NCT04442347	Ib	Active, not recruiting	Arcturus	LUNAR®
mRNA-3927	Propionyl-CoA carboxylase	Propionic acidemia	i.v.	NCT04159103	I/II	Recruiting	Moderna	LNP
NTLA-2001	Cas9	Transthyretin amyloidosis	i.v.	NCT04601051	I	Recruiting	Intellia Therapeutics	LNP
NTLA-2001	Cas9	Transthyretin amyloidosis	i.v.	NCT05697861	Observational	Recruiting	Intellia Therapeutics	LNP
NTLA-2002	Cas9	Hereditary Angioedema	i.v.	NCT05120830	I/II	Recruiting	Intellia Therapeutics	LNP

Note: i.v., intravenous.

## References

[1] Sahin U, Kariko K, Tureci O (2014). mRNA-based therapeutics-developing a new class of drugs. Nat Rev Drug Discov.

[2] Weng Y, Li C, Yang T, Hu B, Zhang M, Guo S (2020). The challenge and prospect of mRNA therapeutics landscape. Biotechnol Adv.

[3] Wu X, Brewer G (2012). The regulation of mRNA stability in mammalian cells: 2.0. Gene.

[4] Grudzien-Nogalska E, Kowalska J, Su W, Kuhn AN, Slepenkov SV, Darzynkiewicz E (2013). Synthetic mRNAs with superior translation and stability properties. Methods Mol Biol.

[5] Wang Y, Su HH, Yang Y, Hu Y, Zhang L, Blancafort P (2013). Systemic delivery of modified mRNA encoding herpes simplex virus 1 thymidine kinase for targeted cancer gene therapy. Mol Ther.

[6] Peng J, Murray EL, Schoenberg DR (2008). *In vivo* and *in vitro* analysis of poly(A) length effects on mRNA translation. Methods Mol Biol.

[7] Kormann MS, Hasenpusch G, Aneja MK, Nica G, Flemmer AW, Herber-Jonat S (2011). Expression of therapeutic proteins after delivery of chemically modified mRNA in mice. Nat Biotechnol.

[8] Thomas CE, Ehrhardt A, Kay MA (2003). Progress and problems with the use of viral vectors for gene therapy. Nat Rev Genet.

[9] Zhang N-N, Li X-F, Deng Y-Q, Zhao H, Huang Y-J, Yang G (2020). A thermostable mRNA vaccine against COVID-19. Cell.

[10] Hussain A, Yang H, Zhang M, Liu Q, Alotaibi G, Irfan M (2022). mRNA vaccines for COVID-19 and diverse diseases. J Control Release.

[11] Hu B, Li B, Li K, Liu Y, Li C, Zheng L (2022). Thermostable ionizable lipid-like nanoparticle (iLAND) for RNAi treatment of hyperlipidemia. Sci Adv.

[12] Li J, Men K, Gao Y, Wu J, Lei S, Yang Y (2021). Single micelle vectors based on lipid/block copolymer compositions as mRNA formulations for efficient cancer immunogene therapy. Mol Pharm.

[13] Qin H, Zhao R, Qin Y, Zhu J, Chen L, Di C (2021). Development of a cancer vaccine using *in vivo* click-chemistry-mediated active lymph node accumulation for improved immunotherapy. Adv Mater.

[14] Medina-Kauwe LK, Xie J, Hamm-Alvarez S (2005). Intracellular trafficking of nonviral vectors. Gene Ther.

[15] Wojnilowicz M, Glab A, Bertucci A, Caruso F, Cavalieri F (2019). Super-resolution imaging of proton sponge-triggered rupture of endosomes and cytosolic release of small interfering RNA. ACS Nano.

[16] Godbey WT, Wu KK, Mikos AG (1999). Tracking the intracellular path of poly(ethylenimine)/DNA complexes for gene delivery. Proc Natl Acad Sci U S A.

[17] Funhoff AM, van Nostrum CF, Koning GA, Schuurmans-Nieuwenbroek NM, Crommelin DJ, Hennink WE (2004). Endosomal escape of polymeric gene delivery complexes is not always enhanced by polymers buffering at low pH. Biomacromolecules.

[18] Benjaminsen RV, Mattebjerg MA, Henriksen JR, Moghimi SM, Andresen TL (2013). The possible "proton sponge " effect of polyethylenimine (PEI) does not include change in lysosomal pH. Mol Ther.

[19] Kim T-i, Kim SW (2011). Bioreducible polymers for gene delivery. React Funct Polym.

[20] Sato Y (2021). Development of lipid nanoparticles for the delivery of macromolecules based on the molecular design of pH-sensitive cationic lipids. Chem Pharm Bull (Tokyo).

[21] Akbarzadeh A, Rezaei-Sadabady R, Davaran S, Joo SW, Zarghami N, Hanifehpour Y (2013). Liposome: classification, preparation, and applications. Nanoscale Res Lett.

[22] Zhang H (2017). Thin-film hydration followed by extrusion method for liposome preparation. Methods Mol Biol.

[23] Has C, Sunthar P (2020). A comprehensive review on recent preparation techniques of liposomes. J Liposome Res.

[24] Michel T, Luft D, Abraham MK, Reinhardt S, Salinas Medina ML, Kurz J (2017). Cationic nanoliposomes meet mRNA: Efficient delivery of modified mRNA using hemocompatible and stable vectors for therapeutic applications. Mol Ther Nucleic Acids.

[25] Zhang R, Tang L, Tian Y, Ji X, Hu Q, Zhou B (2020). DP7-C-modified liposomes enhance immune responses and the antitumor effect of a neoantigen-based mRNA vaccine. J Control Release.

[26] Rosigkeit S, Meng M, Grunwitz C, Gomes P, Kreft A, Hayduk N (2018). Monitoring translation activity of mRNA-loaded nanoparticles in mice. Mol Pharm.

[27] Verbeke R, Lentacker I, Wayteck L, Breckpot K, Van Bockstal M, Descamps B (2017). Co-delivery of nucleoside-modified mRNA and TLR agonists for cancer immunotherapy: Restoring the immunogenicity of immunosilent mRNA. J Control Release.

[28] Kranz LM, Diken M, Haas H, Kreiter S, Loquai C, Reuter KC (2016). Systemic RNA delivery to dendritic cells exploits antiviral defence for cancer immunotherapy. Nature.

[29] He Q, Gao H, Tan D, Zhang H, Wang JZ (2022). mRNA cancer vaccines: Advances, trends and challenges. Acta Pharm Sin B.

[30] Cullis PR, Hope MJ (2017). Lipid Nanoparticle systems for enabling gene therapies. Mol Ther.

[31] Rizk M, Tuzmen S (2017). Update on the clinical utility of an RNA interference-based treatment: focus on Patisiran. Pharmgenomics Pers Med.

[32] Ramachandran S, Satapathy SR, Dutta T (2022). Delivery strategies for mRNA vaccines. Pharmaceut Med.

[33] Kim M, Jeong M, Hur S, Cho Y, Park J, Jung H (2021). Engineered ionizable lipid nanoparticles for targeted delivery of RNA therapeutics into different types of cells in the liver. Sci Adv.

[34] Rak M, Ochalek A, Gawarecka K, Masnyk M, Chmielewski M, Chojnacki T (2020). Boost of serum resistance and storage stability in cationic polyprenyl-based lipofection by helper lipids compositions. Eur J Pharm Biopharm.

[35] Guimaraes PPG, Zhang R, Spektor R, Tan M, Chung A, Billingsley MM (2019). Ionizable lipid nanoparticles encapsulating barcoded mRNA for accelerated *in vivo* delivery screening. J Control Release.

[36] Buschmann MD, Carrasco MJ, Alishetty S, Paige M, Alameh MG, Weissman D (2021). Nanomaterial delivery systems for mRNA vaccines. Vaccines (Basel).

[37] Horiuchi Y, Lai SJ, Yamazaki A, Nakamura A, Ohkawa R, Yano K (2018). Validation and application of a novel cholesterol efflux assay using immobilized liposomes as a substitute for cultured cells. Biosci Rep.

[38] Kim J, Jozic A, Lin Y, Eygeris Y, Bloom E, Tan X (2022). Engineering lipid nanoparticles for enhanced intracellular delivery of mRNA through inhalation. ACS Nano.

[39] Baskararaj S, Panneerselvam T, Govindaraj S, Arunachalam S, Parasuraman P, Pandian SRK (2020). Formulation and characterization of folate receptor-targeted PEGylated liposome encapsulating bioactive compounds from Kappaphycus alvarezii for cancer therapy. 3 Biotech.

[40] Yang Y, Noviana E, Nguyen MP, Geiss BJ, Dandy DS, Henry CS (2017). Paper-based microfluidic devices: Emerging themes and applications. Anal Chem.

[41] Choi A, Seo KD, Kim DW, Kim BC, Kim DS (2017). Recent advances in engineering microparticles and their nascent utilization in biomedical delivery and diagnostic applications. Lab Chip.

[42] Cheng Q, Wei T, Farbiak L, Johnson LT, Dilliard SA, Siegwart DJ (2020). Selective organ targeting (SORT) nanoparticles for tissue-specific mRNA delivery and CRISPR-Cas gene editing. Nat Nanotechnol.

[43] Dilliard SA, Cheng Q, Siegwart DJ (2021). On the mechanism of tissue-specific mRNA delivery by selective organ targeting nanoparticles. Proc Natl Acad Sci U S A.

[44] Bevers EM, Williamson PL (2016). Getting to the outer leaflet: physiology of phosphatidylserine exposure at the plasma membrane. Physiol Rev.

[45] Peng L, Wagner E (2019). Polymeric Carriers for nucleic acid delivery: Current designs and future directions. Biomacromolecules.

[46] Chan LY, Khung YL, Lin CY (2019). Preparation of messenger RNA nanomicelles via non-cytotoxic PEG-polyamine nanocomplex for intracerebroventicular delivery: A proof-of-concept study in mouse models. Nanomaterials (Basel).

[47] Mintzer MA, Grinstaff MW (2011). Biomedical applications of dendrimers: a tutorial. Chem Soc Rev.

[48] Liu S, Wang X, Yu X, Cheng Q, Johnson LT, Chatterjee S (2021). Zwitterionic phospholipidation of cationic polymers facilitates systemic mRNA delivery to spleen and lymph nodes. J Am Chem Soc.

[49] Lallana E (2017). Chitosan/Hyaluronic Acid Nanoparticles: Rational Design Revisited for RNA Delivery. Mol Pharm.

[50] Soliman OY, Alameh MG, De Cresenzo G, Buschmann MD, Lavertu M (2020). Efficiency of chitosan/hyaluronan-based mRNA delivery systems *in vitro*: Influence of composition and structure. J Pharm Sci.

[51] Rejman J, Tavernier G, Bavarsad N, Demeester J, De Smedt SC (2010). mRNA transfection of cervical carcinoma and mesenchymal stem cells mediated by cationic carriers. J Control Release.

[52] Zhang H, De Smedt SC, Remaut K (2018). Fluorescence Correlation Spectroscopy to find the critical balance between extracellular association and intracellular dissociation of mRNA complexes. Acta Biomater.

[53] Lv H, Zhang S, Wang B, Cui S, Yan J (2006). Toxicity of cationic lipids and cationic polymers in gene delivery. J Control Release.

[54] Ishaqat A, Herrmann A (2021). Polymers strive for accuracy: From sequence-defined polymers to mRNA vaccines against COVID-19 and polymers in nucleic acid therapeutics. J Am Chem Soc.

[55] Shen W, Wang Q, Shen Y, Gao X, Li L, Yan Y (2018). Green tea catechin dramatically promotes RNAi mediated by low-molecular-weight polymers. ACS Cent Sci.

[56] Zhao M, Li M, Zhang Z, Gong T, Sun X (2016). Induction of HIV-1 gag specific immune responses by cationic micelles mediated delivery of gag mRNA. Drug Deliv.

[57] Li M, Li Y, Peng K, Wang Y, Gong T, Zhang Z (2017). Engineering intranasal mRNA vaccines to enhance lymph node trafficking and immune responses. Acta Biomater.

[58] Li M, Zhao M, Fu Y, Li Y, Gong T, Zhang Z (2016). Enhanced intranasal delivery of mRNA vaccine by overcoming the nasal epithelial barrier via intra- and paracellular pathways. J Control Release.

[59] Ke X, Shelton L, Hu Y, Zhu Y, Chow E, Tang H (2020). Surface-functionalized PEGylated nanoparticles deliver messenger RNA to pulmonary immune cells. ACS Appl Mater Interfaces.

[60] Gonzalez-Miro M, Rodriguez-Noda L, Farinas-Medina M, Garcia-Rivera D, Verez-Bencomo V, Rehm BHA (2017). Self-assembled particulate PsaA as vaccine against Streptococcus pneumoniae infection. Heliyon.

[61] Ulery BD, Nair LS, Laurencin CT (2011). Biomedical applications of biodegradable polymers. J Polym Sci B Polym Phys.

[62] Jiang Y, Lu Q, Wang Y, Xu E, Ho A, Singh P (2020). Quantitating endosomal escape of a library of polymers for mRNA delivery. Nano Lett.

[63] Karlsson J, Rhodes KR, Green JJ, Tzeng SY (2020). Poly(beta-amino ester)s as gene delivery vehicles: challenges and opportunities. Expert Opin Drug Deliv.

[64] Palmiero UC, Kaczmarek JC, Fenton OS, Anderson DG (2018). Poly(beta-amino ester)-co-poly(caprolactone) terpolymers as nonviral vectors for mRNA delivery *in vitro* and *in vivo*. Adv Healthc Mater.

[65] Moffett HF, Coon ME, Radtke S, Stephan SB, McKnight L, Lambert A (2017). Hit-and-run programming of therapeutic cytoreagents using mRNA nanocarriers. Nat Commun.

[66] Chahal JS, Khan OF, Cooper CL, McPartlan JS, Tsosie JK, Tilley LD (2016). Dendrimer-RNA nanoparticles generate protective immunity against lethal Ebola, H1N1 influenza, and Toxoplasma gondii challenges with a single dose. Proc Natl Acad Sci U S A.

[67] Zhang D, Atochina-Vasserman EN, Lu J, Maurya DS, Xiao Q, Liu M (2022). The unexpected importance of the primary structure of the hydrophobic part of one-component ionizable amphiphilic Janus dendrimers in targeted mRNA delivery activity. J Am Chem Soc.

[68] Mahiny AJ, Dewerth A, Mays LE, Alkhaled M, Mothes B, Malaeksefat E (2015). *In vivo* genome editing using nuclease-encoding mRNA corrects SP-B deficiency. Nat Biotechnol.

[69] Jarzebinska A, Pasewald T, Lambrecht J, Mykhaylyk O, Kuemmerling L, Beck P (2016). A single methylene group in oligoalkylamine-based cationic polymers and lipids promotes enhanced mRNA delivery. Angew Chem Int Ed Engl.

[70] Chen G, Ma B, Wang Y, Gong S (2018). A universal GSH-responsive nanoplatform for the delivery of DNA, mRNA, and Cas9/sgRNA ribonucleoprotein. ACS Appl Mater Interfaces.

[71] Gao X, Huang L (1996). Potentiation of cationic liposome-mediated gene delivery by polycations. Biochemistry.

[72] Chen W, Li H, Liu Z, Yuan W (2016). Lipopolyplex for therapeutic gene delivery and its application for the treatment of Parkinson's disease. Front Aging Neurosci.

[73] Hoerr I, Obst R, Rammensee HG, Jung G (2000). *In vivo* application of RNA leads to induction of specific cytotoxic T lymphocytes and antibodies. Eur J Immunol.

[74] Kaczmarek JC, Patel AK, Kauffman KJ, Fenton OS, Webber MJ, Heartlein MW (2016). Polymer-lipid nanoparticles for systemic delivery of mRNA to the lungs. Angew Chem Int Ed Engl.

[75] Cheng Q, Wei T, Jia Y, Farbiak L, Zhou K, Zhang S (2018). Dendrimer-based lipid nanoparticles deliver therapeutic FAH mRNA to normalize liver function and extend survival in a mouse model of hepatorenal tyrosinemia type I. Adv Mater.

[76] Kowalski PS, Palmiero UC, Huang Y, Rudra A, Langer R, Anderson DG (2018). Ionizable amino-polyesters synthesized via ring opening polymerization of tertiary amino-alcohols for tissue selective mRNA delivery. Adv Mater.

[77] Mockey M, Bourseau E, Chandrashekhar V, Chaudhuri A, Lafosse S, Le Cam E (2007). mRNA-based cancer vaccine: prevention of B16 melanoma progression and metastasis by systemic injection of MART1 mRNA histidylated lipopolyplexes. Cancer Gene Ther.

[78] Van der Jeught K, De Koker S, Bialkowski L, Heirman C, Joe PT, Perche F (2018). Dendritic cell targeting mRNA lipopolyplexes combine strong antitumor T-cell immunity with improved inflammatory safety. ACS Nano.

[79] Kong N, Tao W, Ling X, Wang J, Xiao Y, Shi S (2019). Synthetic mRNA nanoparticle-mediated restoration of p53 tumor suppressor sensitizes p53-deficient cancers to mTOR inhibition. Sci Transl Med.

[80] Xu C, Lu Z, Luo Y, Liu Y, Cao Z, Shen S (2018). Targeting of NLRP3 inflammasome with gene editing for the amelioration of inflammatory diseases. Nat Commun.

[81] Zhao W, Zhang C, Li B, Zhang X, Luo X, Zeng C (2018). Lipid polymer hybrid nanomaterials for mRNA delivery. Cell Mol Bioeng.

[82] Ding Y, Jiang Z, Saha K, Kim CS, Kim ST, Landis RF (2014). Gold nanoparticles for nucleic acid delivery. Mol Ther.

[83] Giljohann DA, Seferos DS, Daniel WL, Massich MD, Patel PC, Mirkin CA (2010). Gold nanoparticles for biology and medicine. Angew Chem Int Ed Engl.

[84] Mendes BB, Conniot J, Avital A, Yao D, Jiang X, Zhou X (2022). Nanodelivery of nucleic acids. Nat Rev Methods Primers.

[85] Erasmus JH, Khandhar AP, O'Connor MA, Walls AC, Hemann EA, Murapa P (2020). An Alphavirus-derived replicon RNA vaccine induces SARS-CoV-2 neutralizing antibody and T cell responses in mice and nonhuman primates. Sci Transl Med.

[86] Mbatha LS, Maiyo F, Daniels A, Singh M (2021). Dendrimer-coated gold nanoparticles for efficient folate-targeted mRNA delivery *in vitro*. Pharmaceutics.

[87] Ho W, Gao M, Li F, Li Z, Zhang X-Q, Xu X (2021). Next-generation vaccines: Nanoparticle-mediated DNA and mRNA delivery. Adv Healthc Mater.

[88] Teixeira HF, Bruxel F, Fraga M, Schuh RS, Zorzi GK, Matte U (2017). Cationic nanoemulsions as nucleic acids delivery systems. Int J Pharm.

[89] Brito LA, Chan M, Shaw CA, Hekele A, Carsillo T, Schaefer M (2014). A cationic nanoemulsion for the delivery of next-generation RNA vaccines. Mol Ther.

[90] Luisi K, Morabito KM, Burgomaster KE, Sharma M, Kong W-P, Foreman BM (2020). Development of a potent Zika virus vaccine using self-amplifying messenger RNA. Sci Adv.

[91] Bogers WM, Oostermeijer H, Mooij P, Koopman G, Verschoor EJ, Davis D (2015). Potent immune responses in Rhesus Macaques induced by nonviral delivery of a self-amplifying RNA vaccine expressing HIV type 1 envelope with a cationic nanoemulsion. J Infect Dis.

[92] Samsa MM, Dupuy LC, Beard CW, Six CM, Schmaljohn CS, Mason PW (2019). Self-amplifying RNA vaccines for venezuelan equine encephalitis virus induce robust protective immunogenicity in mice. Mol Ther.

[93] Alberer M, Gnad-Vogt U, Hong HS, Mehr KT, Backert L, Finak G (2017). Safety and immunogenicity of a mRNA rabies vaccine in healthy adults: an open-label, non-randomised, prospective, first-in-human phase 1 clinical trial. Lancet.

[94] van den Brand D, Gorris MAJ, van Asbeck AH, Palmen E, Ebisch I, Dolstra H (2019). Peptide-mediated delivery of therapeutic mRNA in ovarian cancer. Eur J Pharm Biopharm.

[95] Kurrikoff K, Langel U (2019). Recent CPP-based applications in medicine. Expert Opin Drug Deliv.

[96] Kim Y, Kim H, Kim EH, Jang H, Jang Y, Chi S-G (2022). The potential of cell-penetrating peptides for mRNA delivery to cancer cells. Pharmaceutics.

[97] Udhayakumar VK, De Beuckelaer A, McCaffrey J, McCrudden CM, Kirschman JL, Vanover D (2017). Arginine-rich peptide-based mRNA nanocomplexes efficiently instigate cytotoxic T cell immunity dependent on the amphipathic organization of the peptide. Adv Healthc Mater.

[98] Yokoo H, Oba M, Uchida S (2022). Cell-penetrating peptides: Emerging tools for mRNA delivery. Pharmaceutics.

[99] El Andaloussi S, Maeger I, Breakefield XO, Wood MJA (2013). Extracellular vesicles: biology and emerging therapeutic opportunities. Nat Rev Drug Discov.

[100] Kamerkar S, LeBleu VS, Sugimoto H, Yang S, Ruivo CF, Melo SA (2017). Exosomes facilitate therapeutic targeting of oncogenic KRAS in pancreatic cancer. Nature.

[101] Yang Z, Shi J, Xie J, Wang Y, Sun J, Liu T (2020). Large-scale generation of functional mRNA-encapsulating exosomes via cellular nanoporation. Nat Biomed Eng.

[102] Yang D, Zhang W, Zhang H, Zhang F, Chen L, Ma L (2020). Progress, opportunity, and perspective on exosome isolation - efforts for efficient exosome-based theranostics. Theranostics.

[103] Fu X, Chen T, Song Y, Feng C, Chen H, Zhang Q (2021). mRNA delivery by a pH-responsive DNA nano-hydrogel. Small.

[104] Nitika Wei J, Hui A-M (2022). The delivery of mRNA vaccines for therapeutics. Life (Basel).

[105] Kaczmarek JC, Patel AK, Rhym LH, Palmiero UC, Bhat B, Heartlein MW (2021). Systemic delivery of mRNA and DNA to the lung using polymer-lipid nanoparticles. Biomaterials.

[106] Parhiz H, Shuvaev VV, Pardi N, Khoshnejad M, Kiseleva RY, Brenner JS (2018). PECAM-1 directed re-targeting of exogenous mRNA providing two orders of magnitude enhancement of vascular delivery and expression in lungs independent of apolipoprotein E-mediated uptake. J Control Release.

[107] Karlsson J, Luly KM, Tzeng SY, Green JJ (2021). Nanoparticle designs for delivery of nucleic acid therapeutics as brain cancer therapies. Adv Drug Deliv Rev.

[108] Tombacz I, Laczko D, Shahnawaz H, Muramatsu H, Natesan A, Yadegari A (2021). Highly efficient CD4+T cell targeting and genetic recombination using engineered CD4+cell-homing mRNA-LNPs. Mol Ther.

[109] Homayun B, Lin X, Choi H-J (2019). Challenges and recent progress in oral drug delivery systems for biopharmaceuticals. Pharmaceutics.

[110] Sahin U, Kariko K, Tuereci O (2014). mRNA-based therapeutics - developing a new class of drugs. Nat Rev Drug Discov.

[111] Zhang X, Men K, Zhang Y, Zhang R, Yang L, Duan X (2019). Local and systemic delivery of mRNA encoding survivin-T34A by lipoplex for efficient colon cancer gene therapy. Int J Nanomedicine.

[112] Liang F, Lindgren G, Lin A, Thompson EA, Ols S, Rohss J (2017). Efficient targeting and activation of antigen presenting cells *in vivo* after modified mRNA vaccine administration in Rhesus Macaques. Mol Ther.

[113] Turner JS, O'Halloran JA, Kalaidina E, Kim W, Schmitz AJ, Zhou JQ (2021). SARS-CoV-2 mRNA vaccines induce persistent human germinal centre responses. Nature.

[114] Pardi N, Hogan MJ, Porter FW, Weissman D (2018). mRNA vaccines - a new era in vaccinology. Nat Rev Drug Discov.

[115] Anderluzzi G, Lou G, Woods S, Schmidt ST, Gallorini S, Brazzoli M (2022). The role of nanoparticle format and route of administration on self-amplifying mRNA vaccine potency. J Control Release.

[116] Rini BI, Stenzl A, Zdrojowy R, Kogan M, Shkolnik M, Oudard S (2016). IMA901, a multipeptide cancer vaccine, plus sunitinib versus sunitinib alone, as first-line therapy for advanced or metastatic renal cell carcinoma (IMPRINT): a multicentre, open-label, randomised, controlled, phase 3 trial. Lancet Oncol.

[117] Ibrahim MM (2010). Subcutaneous and visceral adipose tissue: structural and functional differences. Obes Rev.

[118] Gradel AKJ, Porsgaard T, Lykkesfeldt J, Seested T, Gram-Nielsen S, Kristensen NR (2018). Factors affecting the absorption of subcutaneously administered insulin: Effect on variability. J Diabetes Res.

[119] Lobaina Mato Y (2019). Nasal route for vaccine and drug delivery: Features and current opportunities. Int J Pharm.

[120] Zhuang X, Qi Y, Wang M, Yu N, Nan F, Zhang H (2020). mRNA vaccines encoding the HA protein of influenza A H1N1 virus delivered by cationic lipid nanoparticles induce protective immune responses in mice. Vaccines (Basel).

[121] Mai Y, Guo J, Zhao Y, Ma S, Hou Y, Yang J (2020). Intranasal delivery of cationic liposome-protamine complex mRNA vaccine elicits effective anti-tumor immunity. Cell Immunol.

[122] Li JQ, Zhang ZR, Zhang HQ, Zhang YN, Zeng XY, Zhang QY (2021). Intranasal delivery of replicating mRNA encoding neutralizing antibody against SARS-CoV-2 infection in mice. Signal Transduct Target Ther.

[123] Dhaliwal HK, Fan Y, Kim J, Amiji MM (2020). Intranasal delivery and transfection of mRNA therapeutics in the brain using cationic liposomes. Mol Pharm.

[124] Heida R, Hinrichs WL, Frijlink HW (2022). Inhaled vaccine delivery in the combat against respiratory viruses: a 2021 overview of recent developments and implications for COVID-19. Expert Rev Vaccines.

[125] de Bree GJ, van Leeuwen EM, Out TA, Jansen HM, Jonkers RE, van Lier RA (2005). Selective accumulation of differentiated CD8+ T cells specific for respiratory viruses in the human lung. J Exp Med.

[126] Jiang AY, Witten J, Raji IO, Eweje F, MacIsaac C, Meng S (2023). Combinatorial development of nebulized mRNA delivery formulations for the lungs. Nat Nanotechnol.

[127] Zhang H, Leal J, Soto MR, Smyth HDC, Ghosh D (2020). Aerosolizable lipid nanoparticles for pulmonary delivery of mRNA through design of experiments. Pharmaceutics.

[128] Patel S, Ryals RC, Weller KK, Pennesi ME, Sahay G (2019). Lipid nanoparticles for delivery of messenger RNA to the back of the eye. J Control Release.

[129] Tanaka H, Nakatani T, Furihata T, Tange K, Nakai Y, Yoshioka H (2018). *In vivo* introduction of mRNA encapsulated in lipid nanoparticles to brain neuronal cells and astrocytes via intracerebroventricular administration. Mol Pharm.

[130] Samaridou E, Heyes J, Lutwyche P (2020). Lipid nanoparticles for nucleic acid delivery: Current perspectives. Adv Drug Deliv Rev.

[131] Zhang M, Hussain A, Yang H, Zhang J, Liang X-J, Huang Y (2023). mRNA-based modalities for infectious disease management. Nano Research.

[132] Polack FP, Thomas SJ, Kitchin N, Absalon J, Gurtman A, Lockhart S (2020). Safety and efficacy of the BNT162b2 mRNA Covid-19 vaccine. N Engl J Med.

[133] Barda N, Dagan N, Balicer RD (2021). BNT162b2 mRNA Covid-19 vaccine in a nationwide mass vaccination setting. Reply. N Engl J Med.

[134] Chen GL, Li XF, Dai XH, Li N, Cheng ML, Huang Z (2022). Safety and immunogenicity of the SARS-CoV-2 ARCoV mRNA vaccine in Chinese adults: a randomised, double-blind, placebo-controlled, phase 1 trial. Lancet Microbe.

[135] Husain M (2014). Avian influenza A (H7N9) virus infection in humans: epidemiology, evolution, and pathogenesis. Infect Genet Evol.

[136] Zhang C, Maruggi G, Shan H, Li J (2019). Advances in mRNA vaccines for infectious diseases. Front Immunol.

[137] Feldman RA, Fuhr R, Smolenov I, Mick Ribeiro A, Panther L, Watson M (2019). mRNA vaccines against H10N8 and H7N9 influenza viruses of pandemic potential are immunogenic and well tolerated in healthy adults in phase 1 randomized clinical trials. Vaccine.

[138] Schnee M, Vogel AB, Voss D, Petsch B, Baumhof P, Kramps T (2016). An mRNA vaccine encoding Rabies virus glycoprotein induces protection against lethal infection in mice and correlates of protection in adult and newborn pigs. PLoS Negl Trop Dis.

[139] Aldrich C, Leroux-Roels I, Huang KB, Bica MA, Loeliger E, Schoenborn-Kellenberger O (2021). Proof-of-concept of a low-dose unmodified mRNA-based rabies vaccine formulated with lipid nanoparticles in human volunteers: A phase 1 trial. Vaccine.

[140] Gray GE, Bekker LG, Laher F, Malahleha M, Allen M, Moodie Z (2021). Vaccine efficacy of ALVAC-HIV and bivalent subtype C gp120-MF59 in adults. N Engl J Med.

[141] Zhang P, Narayanan E, Liu Q, Tsybovsky Y, Boswell K, Ding S (2021). A multiclade env-gag VLP mRNA vaccine elicits tier-2 HIV-1-neutralizing antibodies and reduces the risk of heterologous SHIV infection in macaques. Nat Med.

[142] Bialas KM, Permar SR (2016). The March towards a vaccine for congenital CMV: Rationale and models. PLoS Pathog.

[143] John S, Yuzhakov O, Woods A, Deterling J, Hassett K, Shaw CA (2018). Multi-antigenic human cytomegalovirus mRNA vaccines that elicit potent humoral and cell-mediated immunity. Vaccine.

[144] Sahin U, Oehm P, Derhovanessian E, Jabulowsky RA, Vormehr M, Gold M (2020). An RNA vaccine drives immunity in checkpoint-inhibitor-treated melanoma. Nature.

[145] Terheyden P, Weishaupt C, Heinzerling L, Klinkhardt U, Krauss J, Mohr P (2018). Phase I dose-escalation and expansion study of intratumoral CV8102, a RNA-based TLR- and RIG-1 agonist in patients with advanced solid tumors. Annals of Oncology.

[146] Patel MR, Bauer TM, Jimeno A, Wang D, LoRusso PM, Do KT (2020). A phase I study of mRNA-2752, a lipid nanoparticle encapsulating mRNAs encoding human OX40L, IL-23, and IL-36γ, for intratumoral (iTu) injection alone and in combination with durvalumab. Journal of Clinical Oncology.

[147] Billingsley MM, Singh N, Ravikumar P, Zhang R, June CH, Mitchell MJ (2020). Ionizable lipid nanoparticle-mediated mRNA delivery for human CAR T cell engineering. Nano Lett.

[148] Rurik JG, Tombácz I, Yadegari A, Méndez Fernández PO, Shewale SV, Li L (2022). CAR T cells produced *in vivo* to treat cardiac injury. Science.

[149] Gillmore JD, Gane E, Taubel J, Kao J, Fontana M, Maitland ML (2021). CRISPR-Cas9 *in vivo* gene editing for transthyretin amyloidosis. N Engl J Med.

[150] Xie W, Chen B, Wong J (2021). Evolution of the market for mRNA technology. Nat Rev Drug Discov.

